# Clinical Review, and Hospital Reports

**Published:** 1837-01-01

**Authors:** 


### Clinical Review.

### Guy's Hospital.

Guy's Hospital Reports. No. II, for April, 1836, and No. III, for September, 1836. Edited by L. H. Barlow, M. A. &c. &c. &c., and J. P. Babington, M.A. &c. &c.

These Reports continue to be published with a spirit equally honourable to the medical officers of the institution, and to the editors themselves. We hope that the publication will be persisted in, for the facts are valuable, and their authenticity undoubted. We shall take care that the principal are more widely disseminated than they can be in their original form, and that the Physicians and Surgeons of Guy's Hospital shall obtain the credit which they merit—that of taking the lead among their corps in rendering the stores of information contained within the hospital walls, available, as far as possible, to the whole profession.

We have fallen into arrears with these Reports, and two numbers lie on the table before us. The second number has been scarcely noticed, and the only article which we have taken from the third, is contained in our present Periscope.*

In this large mass of reports the difficulty consists in selection. We shall notice those which appear to us to possess most immediate and practical interest, or which bear on some intricate or curious question.

I. Some Observations on the Nature and Treatment of Ganglion, Bunion, &c. &c. By Mr. Aston Key.

Mr. Key seems to doubt whether a ganglion is a true bursa mucosa, because it is found where naturally bursæ do not exist. The fact, however, is that the pathology of the bursæ themselves is by no means clear, for they appear to be capable of being developed in the common cellular tissue, in spots where, naturally, they are not to be found. Thus they have been found on the ends of a bone after amputation, covering exostoses, and so on. Nor are the contents of bursæ mucosæ of uniform character. In one case we have little more than a sort of serous exhalation, the fluid being barely albuminous—in another we have a thick glairy fluid—in a third, we have a fluid mixed with the "melon-seed bodies." Amidst these varieties it is difficult to determine whether a ganglion is or is not a bursa mucosa, for both seem to spring up under nearly similar circumstances, and both seem to be frequently developed from the common cellular membrane. Be this as it may, it is our object to cure a ganglion. Let us hear what Mr. Key has to say upon this point.

"The treatment usually adopted for those situated on the back of the wrist-joint, is, to burst the cyst by a sharp blow with a book or any other hard body. If well aimed and sharply struck, it often effects the object of breaking it down; but it as frequently fails, either from the firmness of the cyst, or from the unskilfulness of the blow, or from want of sufficient resistance afforded by the wrist. It is also a painful remedy, and is occasionally productive of more subsequent inconvenience than the ganglion had occasioned. The more simple plan is, that of making a small puncture with the point of a lancet; or a cataract-needle, when the tumor is small. There is no fear to be entertained of inflammation taking place; as the cyst is little disposed to inflame, and the closure of the opening, after the contents have escaped, by means of a band or piece of plaster, is a sufficient guard against its occurrence. I have never seen or known any mischief to arise from the operation of puncturing these ganglia; and their return is equally prevented as by any other mode of treatment.

* Experiments of Sir A. Cooper on the vertebral and carotid arteries.

For the small tumors that occupy the base of the palmar side of the fingers, the puncture is the only remedy. Blisters or blows are ineffectual: the former are very slow in operating, and rarely succeed in removing them; and blows cannot be struck with sufficient accuracy to ensure a rupture of the cyst. The cataract-needle is the most convenient remedy: the tumor being sensitive, the patient experiences some pain in its introduction; but it is momentary, and the relief afforded is complete.

When the swelling is situated over the back of a joint on the extensor tendon, pressure should be kept up for some weeks after the evacuation of the cyst. A gentleman, who was much in the habit of writing, applied to me for a swelling of this kind on the first phalangeal joint of the right index: he complained of pain and weakness in the use of it, and was unable to employ the finger steadily in writing. A small puncture was made in it, and about a drachm of crystalline fluid escaped: a piece of ribband was bound over the joint, and firm pressure was thus made on the cyst. The fluid did not collect again; and by wearing the ribband for a few weeks, in order to secure and steady the finger when employed in writing, he regained the perfect use and strength of it.

I once punctured a similar swelling over the tendon of the trochlearis superior muscle of the eye; and it never shewed any disposition to return.—Occasionally, the part that has been the seat of the ganglionic enlargement becomes thickened after its contents have been allowed to escape; and a slight hardness remains, but does not interfere with the use of the part."

We fear that Mr. Key does not say very much in favour of the puncturing ganglia, when he tells us it is as successful as any other plan of treatment. The fact is, that so far as we have seen neither bursting nor puncturing them is, in general, successful. We have frequently tried both methods, but rarely with the effect of obtaining a permanent cure. On the whole we prefer puncturing, but the young surgeon must not anticipate a great deal from it. Fortunately the complaint is generally little more than an inconvenience. We have several times seen a ganglion form over the tendon of the tibialis anticus near its insertion. It is troublesome, in consequence of the pressure of the boot. Puncturing and subsequent pressure succeeded in the case of a gentleman who was lately under our care.

*Enlarged Bursa over the Patella.*

Every student knows this as the "house-maid's knee." For a slight case pressure with a good knee cap is sufficient. If the bursa becomes inflamed, we must leech and blister, and use pressure subsequently.

Supposing that the bursa is large and troublesome, which it rarely is, unless the cyst is decidedly thickened, pressure is then insufficient for its cure. Mr. Key in this case recommends the employment of a seton, which, he says, is productive of less annoyance to the patient than a blister.

"If a few threads be passed through a ganglion patellæ of one knee, and a blister applied to another on the opposite limb, the patient invariably, I have noticed, complains of more suffering from the blistered surface than from the suppuration caused by the seton: and this is in accordance with what we know of the sensitiveness of the skin, compared with the sensibility of a bursa, even in a state of inflammation. The pain complained of during the stage of acute inflammation is inconsiderable; and the moving the threads, during the process of suppuration, occasions but little uneasiness. Sometimes a little feverish excitement is produced by the pus not escaping freely from the apertures, which, being retained, becomes decomposed: this inconvenience is remedied by keeping the openings, where the silk enters, free and clear. The seton should be kept in for several days after suppuration is established, in order to promote granulations, and to facilitate the escape of the pus. Even where the ganglion suppurates of itself, the seton may be used with advantage, towards accomplishing these two objects."

When the cyst is decidedly thickened, the tumor of any size, and the inconvenience such as to make a patient desirous of something decisive being attempted, we agree with Mr. Key that blisters are very troublesome, and very uncertain. The choice of remedies lies in such a case between the seton and incision. The former is the milder remedy, the latter is the surer, and we think it would be usually the quicker one. If the bursa is laid open, and the cavity dressed in with lint, we may feel confident that it will be totally obliterated. But perhaps the seton would not offer the same degree of certainty. In hydrocele, where, indeed, the sac is generally larger, the seton is not so efficient as incision. The selection of the one method or the other must, after all, be left to the judgment of the surgeon, and depend on the circumstances of the case.

When the bursa has been enlarged for some time, has been subjected to pressure, and has been repeatedly attacked with slight or with severe inflammation, the cyst becomes extraordinarily thickened, the cavity much diminished, and frequently in their interior are seen those "melon-seed" bodies, evidently fibrinous, which have frequently attracted the notice of surgeons. For the benefit of our younger readers we extract a short descriptive passage from the report.

"These hardened ganglia cause great uneasiness to the subject of them; and incapacitate the person from bearing on the part in kneeling, as well as weaken the limb in the act of extension. The appearance of these tumors, when they are laid open, sufficiently indicates their manner of growth, and also the mode in which the loose bodies, resembling melon-seeds, that are often found mixed with their more fluid contents, are produced. The drawing, *Fig*. 5, shews a section of one of these indurated swellings: the size of the cavity is often small in proportion to the whole mass of the swelling; the principal part of which is made up of the thickened parietes. These are seen to consist of layers of condensed fibrine, resembling ligamentous structure, and joined together by a similar tissue. The cavity presents the latest deposits in various forms, but all partaking of the nature of fibrine: in one part are seen shreds of adhesive matter, as if recently effused, adhering at different points of the cyst: in other parts, the effused fibrine assumes a more globular form, with thin peduncular attachments. It is these that give rise to the melon-seed bodies, that may often be seen, in large numbers, floating in the serum. Motion, friction, and pressure give them the flattened ovoid figure; and the peduncles, being thin, break, and allow them to escape from their attachments, and to become loose bodies."

From Mr. Key's description we should be led to suppose, that these bodies are only found in bursæ with greatly thickened parietes. This, however, is not the case. We have seen them in the suprà patellar bursa, when its walls were very little thickened, and they are not uncommonly seen in enlarged bursæ about the wrist and over the inferior angle of the scapula, when the cyst is even thin.

Mr. Key observes that the operation usually performed for such indurated cysts on the patella or its ligament, is excision. He recommends the seton where the tumor is large. It appears to us, from the cases we have seen, that the seton is scarcely applicable to those to which excision is. The latter is adapted for the bursa when it is very indurated, and the cavity almost obliterated. The seton or incision is more appropriate where there is still a cavity, and the walls are thickened. If either incision or the seton were employed, where the cyst has almost become converted, as it occasionally is, into a solid tumor, we should conceive that the cure would be very tedious at the best.

Mr. Key takes no notice of an accidental consequence of enlarged patellar bursæ which we have witnessed several times. While the parietes of the bursa are yet thin, it becomes, from some cause or other, inflamed, and the inflammation extends to the surrounding cellular tissue. Suppuration occurs both in the bursa, and on the outside of it, and, in some instances, ulceration lays open the bursa into the suppurating cavity around it. The case looks formidable. The knee is much swollen, the integument is red, there is fluctuation, the joint is very painful, and there is pyrexia. An inexperienced surgeon would suppose there was suppuration in the synovial cavity—an experienced surgeon, of course would not. Incisions properly directed, evacuate the pus, and are generally successful. In one instance we saw the patient, an elderly female, sink.

It must not be supposed that the seton is always a safe remedy. Mr. Key does not allude to the risk that may follow its employment in some cases of enlarged bursa.* Yet it is right that surgeons should be aware of its occasional dangerous effects. It is not very uncommon for the bursa situated over the inferior angle of the scapula to become enlarged. In a case in St. George's Hospital, in which Mr. Walker passed a seton through this bursa, inflammation and suppuration ensued in the surrounding cellular membrane, the shoulder-joint became implicated, matter formed in it, and the patient died from the violent constitutional disturbance.

In another case at the same hospital, low fever succeeded the introduction of the seton, and the patient was destroyed by purulent deposits in the lungs.

We have heard of another case in private practice in which the same operation was fatal: and we saw a patient *nearly* die from the introduction of a seton into one of the *vaginal* bursæ on the dorsum of the wrist. Diffuse inflammation of the cellular membrane of the fore-arm followed, and, as we mentioned, the patient nearly died. Of course the more strictly subcutaneous the bursa is, the less risk will there be from the use of the seton.

* It should be observed in reference to this and some other apparent omissions on Mr. Key's part, that he is speaking rather of thickened bursa, or ganglion, than of enlarged bursæ, in general.—*Eds.*

*Distortion of the Foot from Pressure.*

Mr. Key makes some remarks on the inconveniences and distortions resulting from undue pressure on some parts of the tarsus.

The first deformity he describes is that which resulted from the use of boots so short, that they compressed the foot in the direction of its length. The foot had been thus made to assume a curved direction: the arch of the tarsus had been considerably increased, producing a large projection on its dorsum, and a corresponding hollow in the sole, rendering the foot altogether shorter than nature had intended it to be. The altered form, however, was the least part of the inconvenience; for the act of progression had become uneasy and cramped. By the unnatural strain created upon the top of the arch at the junction of the os naviculare and os cuneiforme internum, the ligaments had been inflamed, and had become thickened; not, as in the case of the patella, forming a bursal cavity, but giving rise to a painful and indurated swelling, which gave pain under the pressure of the boot, and rendered the foot stiff and uneasy in the act of progression.

2. "A remarkable instance of this kind of deformity, but carried to a greater extent than in the former case, occurred in a boy who was admitted into the hospital with a sore on the ball of the great toe. The lad had been observed to walk lame for a great length of time, in consequence of a small sore, which had resisted all the ordinary means of treatment under the hands of an experienced country practitioner. A lady of the village where he resided obtained admission for him into the hospital. The sore on his foot was a circular foul ulcer on the ball of the great toe; not deep, but of an unhealthy character. The shape of the foot immediately struck the eye as being peculiar and somewhat deformed. The tarsus was more arched than usual; and the phalanges of the toes formed angles with the metatarsal bones, being thrown upward so as to make the balls of the toes more than usually prominent. The great toe was very much distorted upward; and the ball was thus rendered so prominent as to sustain much inconvenience in walking: the result was, that the skin became inflamed, and at length ulcerated. The change of shape which the end of the foot has undergone is at once perceived; and, by the boy's account, is clearly referrible to the undue pressure occasioned by his shoes. His father, in order to make his shoes serviceable, had them cased with iron round the edge of the sole. The effect of this was, that the shoes certainly did not wear out; but his feet, as they enlarged, were obliged to regulate their growth by the form of their iron case; and after he had worn his shoes twelve months, his feet became too large for them, and he limped from the pain which the compression occasioned.—His father compelled him, however, to wear his shoes; but could not prevent the feet growing; and the end of his foot assumed the form which the sketch exhibits."

In another instance small and short shoes caused similar distortion of the foot, and an obstinate deep ulcer at the under part of the ball of the great toe. On examination Mr. Key discovered one of the sesamoid bones exposed. He removed it, and, under a lotion of kreosote, the wound healed.

*Bunion.*

Mr. Key's description of the phases of bunion is more precise, and probably, more correct, than those we generally meet with. Bunion is usually considered to be the mere effect of pressure on the prominence of the metatarso-phalangeal joint of the great toe. Yet a cursory inspection of a foot ornamented with a bunion might lead us to suspect that, in many cases at all events, there is something more than this. Mr. Key's researches into the pathology of bunions tend to verify the suspicion in question.

The foot, we all know, is a double arch. Behind it rests on the inferior tubercles of the calcaneum—before on the phalangeal ends of the metatarsal bones. It does not, however, bear equally on all these, for the central ones are in some measure elevated from the ground, and the chief points of support are metatarsal bones of the great and the little toe. Thus we have a sort of arch extending from side to side at the distal ends of the metatarsal bones, in addition to the arch from before backwards, found between these and the calcaneum. In front of the metatarsal bones we have the toes, themselves rather curved as to be concave inferiorly, and extending the basis of the support, when, the heel being raised from the ground in progression, the great tarso-metatarsal arch no longer sustains the super-incumbent weight.

Such is the natural state of the foot, so far as the disposition of the bones is concerned. Occasionally that state is altered, and one of its alterations gives rise to bunion. Let us listen to our author.

"In young persons, who are allowed or forced to take exercise on foot, disproportioned to their strength, an alteration first ensues in the form of the tarsal arch. It becomes flattened, in consequence of the ligaments, and probably the bones also, yielding to the superincumbent weight. If the pressure be continued, the under part of the arch at length nearly comes in contact with the ground, and the patient becomes flat-footed. On the outer part of the tarsus a corresponding change is perceptible: its upper surface becomes depressed and hollow, and thus a general appearance of deformity and awkwardness of gait are the result. I have seen the young among the female part of many families gradually acquiring this tarsal deformity, from the excess of exercise which they were enjoined by their parents to take; and, in truth, while the error of our forefathers led them to confine girls within too strict limits, and to allow them too little use of their limbs, that of the present day runs into the opposite extreme; and many a young person has the proportions of her feet spoiled, and the foundation of bunion laid, by being kept on her feet for hours without rest or intermission. In some, the deformity is confined to a slight projection of the head of the astragalus, which has weighed down the inner strong plantar ligament. This gives an uncouthness to the walk of the girl, and renders the foot unsightly, but produces no other effect. The change of shape is not long confined to the tarsus; the joints of the toes suffer next, especially that of the great toe. The ligaments of this joint being strained in common with those of the tarsus, at length yield, and the toe is gradually forced outwards by the oblique bearing of the foot on its inner plantar surface. It is rarely that we find persons whose tarsal arch is flattened with a great toe in a line with the foot: it is usually inclined outwards, so as to form an angle with the metatarsal bone. The inner part of the joint thus forms an angular projection; slight at first, but becoming more and more prominent, as the pressure increases, and the joint continues to yield."

This kind of deformity in the arch of the foot leads to bunion. The lateral ligament pressed by the edge of the base of the phalanx becomes thickened and painful, and between its layers a series of small bursal cysts are found. As soon as one of these cavities is obliterated by inflammation, another is formed; and thus, by their successive formation, the effects of pressure are warded off. In some instances, the irritation extends as far as the bone, and a fungous growth takes place from the cartilage. So long as these cysts remain in the state here represented, but little inconvenience is experienced; the tendinous structure has adapted itself to the increased pressure it has to sustain, and the absence of inflammation exempts the person from pain. The pressure of the shoe, however, is frequently followed by paroxysms of severe suffering; the part becoming the seat of inflammatory action, and unable to bear the slightest pressure, either from without, or from the base of the phalanx in progression. Sometimes an abscess occurs on the most projecting part of the swelling, the pain attending which is most severe; and, from the unyielding nature of the texture, the matter is slow to discharge itself by the process of ulceration.

The treatment of bunion is of some little consequence; for it is a troublesome complaint enough. In the early stage more can be effected than at a later period. When the arch of the foot first begins to yield, and pain is experienced in walking, it is high time for the young lady to be transferred from the dancing-master to the surgeon.

"I have tried," says Mr. K., "some plans for the relief of the sufferer, and for the prevention of that greatest of annoyances, a painful bunion; and the best that I have contrived is the following. The offending toe is placed in a separate compartment of the stocking, like the finger of a glove: this again is enclosed in a separate part of the shoe, which is contrived by fixing a piece of firm cow-leather in the sole of the shoe so as to form a separate apartment for the toe. By these means it is kept in a straight line with the foot, or parallel to its fellows; and the pressure against the inner side of the joint being removed, the joint acquires a sufficient degree of strength, to enable it, in a few months, to dispense with the artificial support.

The common plan resorted to for the relief of bunion palliates the evil, in some degree, by removing the pain, and taking off the pressure; but it does not go to the root of the evil. The plasters on soft thick leather are agreeable to a painful bunion, by keeping the skin in a pliant state, and by protecting the part, in some measure, from the pressure of the shoe. The plan above explained effects both these purposes more completely, and prevents the further disposition to the disease, by correcting the inclination of the great toe. I have tried it in many instances of extreme pain; and have found the patient able to walk well, when provided with the shoe."

When an abscess has formed it is better not to be in a hurry to open it. If punctured too early, inflammation is apt to extend into the joint, and to give rise to tedious disease of the bone. Mr. K. has known gangrene of the foot, and death, ensue from opening an inflamed and suppurating bunion; and, in three cases, exfoliation of the bones, with a most tedious and painful suppuration of the surrounding structures. It is better to let Nature carry on the operation, without interfering with the knife: the abscess is slow in coming to the surface; but the joint beneath is secured; and we often have the satisfaction of finding that, the suppurative process being at an end, the sore heals, and the part loses its former extreme sensibility.

---

### Nottingham General Hospital.

### Spasms of the Muscles of the Neck.

Under this head Dr. Hutchinson has related some cases in our contemporary the Lancet, which are deserving of notice. They are considered by Dr. H. as exemplifying irritation of the nervous accessories or par vagum, arising at the base of the brain itself, or in the upper part of the medulla spinalis—" or indirectly reflected from disordered function in distant organs—especially the stomach." The most prominent symptom was spasm, more or less constant, of the trapezius muscles, or the sterno-cleido mastoideus. In one of the three cases, both trapezii were violently contracted. The spasm is very painful, and increased by any exercise. Perfect quietude was the sufferer's own choice. The sleep was undisturbed while it continued. The spasms continued through the day, with little intermission. The pulse was little affected. The appetite good; but the stomach was disordered. Two of the three cases occurred in sedentary and relaxed males—the third was a robust agriculturer of hard-working habits.

*Case* 1. A stocking-weaver, aged 60, pale and unhealthy, long subject to dyspepsy, was admitted into the Nottingham General Hospital on the 24th March, for wandering pains in the head, giddiness, impaired vision. The pains, which appeared neuralgic, extended down the neck. Appetite bad, tongue furred—bowels regular—pulse feeble, and rather increased in frequency—flatulence. Aperient medicines, &c. were ordered. On the 5th of April, the spasmodic affection already described came on, and continued, with more or less violence, till he left the hospital on the 24th June, when he was much relieved, but not cured. Indeed it was ascertained that he had as violent returns as ever, but he was lost sight of, and the event is unknown. Various remedies were employed, especially the carbonate of iron in herculean doses, with aperients, the shower-bath—blisters—counter-irritants of croton oil—leeches, &c. The counter-irritation appeared to produce more benefit than any other remedy.

That this was a neuralgic affection connected with, or depending on, disordered digestion, there can be little doubt amongst practical observers.—Why the sympathetic irritation should locate itself in one particular nerve, or class of nerves, in preference to others, it is often difficult or impossible to say. But in several instances, we thought we discovered the cause of the predisposition, namely, in exposure of the part affected to the influence of climate more than other parts of the body. We suspect that this is the most common cause of the morbid predisposition, especially in neuralgic and rheumatic affections.

*Case* 2. J. Turner, aged 30, an agricultural labourer, of athletic form, and florid complexion, was admitted on the 2d of December last. Six months previously he became affected with stiffness and pain in the back of the neck, extending to the head. The pain was of a darting, stabbing kind, with heat, and twitching of the trapezius muscle of the left side. This has continued all the six months, during which he was under the care of a highly respectable surgeon at Mansfield. It appeared that his general health, though now restored, was much disordered three months previously. The bowels are much confined—the tongue furred—complexion and eyes *yellow*—urine high-coloured—pulse 84—left trapezius tight as a cord, in which the sternocleido of that side participates. The carbonate of iron (ʒij. ter die) was ordered, with pil. col. comp.—Electricity. There was no improvement.—Acupuncture and increased doses of the subcarbonate. Relieved for a short time, when the spasms became more violent than ever, though the pain was less. By the 13th of December, he was much improved under the same treatment. The twitchings were as violent as ever. The veratria ointment was applied, and with apparent relief. On the 17th, however, we find him as bad as ever, or worse. The right pupil is dilated—pulse feeble and frequent—bowels regular—no rigor. The veratria ointment produced an eruption, attended with great heat. Had four grains of calomel and a dose of oil of turpentine. Slept better, but not at all relieved. Complains of pain (for the first time) in the head. Cupped to 14 ounces. 19th. The paroxysms are now more violent at stated hours—generally at 12 and 5 o'clock. *Was freely purged—relieved by the cupping—dilatation of pupil gone—pulse less frequent. Has omitted the iron since the 17th—sleeps better—painless.* Takes small doses of liq. arsenicalis and laudanum. On the 27th we find the pain of head returned. Was cupped—the arsenic discontinued, as the periodicity of the attacks has been removed—purgation. The spasms remaining undiminished, the head was shaved—moxa was applied on each side of the cervical vertebra—soda and hydrocyanic acid ter die—colocyth pills—shower bath, at first tepid, afterwards cold. This treatment was continued through all January (1836) with some improvement. In February and March he lived generously, pursuing the same medicinal treatment, with the addition of quinine. He was discharged cured early in April.

It may be proper to state, that the patient, and indeed the physician, ascribes the great increase of the complaint, on the 17th December, to cold.

*Case* 3. This was a stocking-weaver, of unhealthy aspect, who had had the complaint for four months before he came under our author's care.—Purgatives, the carbonate of iron, the moxas—blisters—iodine externally, &c. were the chief remedies employed for six or seven weeks, when he left the hospital at his own request, relieved, but not cured.

Excepting the peculiar *locale* of the complaint, there does not appear to be any essential difference between it and neuralgic affections in general. Spasms are by no means unfrequent accompaniments of neuralgia in other muscles, and especially long muscles. Twitchings are very frequent, or rather constant attendants on tic douloureux.—Without disputing, in the slightest degree, the propriety of Dr. Hutchinson's *methodus medendi,* we may be permitted to observe that, in our own practice, we have derived more benefit from pretty free and continued purgation, with smart doses of colchicum, than from steel or other tonics, especially at the beginning. The success of the treatment in the foregoing cases does not hold out any very great encouragement to other practitioners. Indeed Dr. H. himself does not seem to be very well satisfied with the results, when he informs us that, should another case occur, he will divide the *nervus accessorius.*—We have little faith in this *anceps remedium,* but sincerely wish Dr. H. success in its employment.

---

### On Several Affections of the Liver.

### 1. Observations on Fatty Degeneration of the Liver. By Dr. Addison.*

Dr. Addison observes that so little is known, both of the nature and the symptoms of this affection of the liver, that any communications which tend to augment our information with respect to it are exceedingly desirable. Dr. Addison, whose zeal and abilities are well known, details the results of his own observations.

The appearance of a liver which presents the fatty degeneration, is described by Dr. A. in the following terms.

"It is observed to be of a cream or pale-yellow colour, figured irregularly

* Guy's Hospital Reports.

with brownish or deep-orange spots. It is usually, though not always, more or less enlarged, and sometimes very considerably so.

When cut into, its interior is found to present an appearance somewhat corresponding to that of the exterior; excepting that the brown and pale-yellow tissues are much more uniformly distributed throughout the entire substance of the organ than they are upon its surface. It is sometimes softer, and more readily crushed between the fingers than is the healthy liver: sometimes, however, it is firmer than natural, and occasionally even of a scirrhous or almost horny hardness. It will often very perceptibly grease the scalpel, or impart an unctuous feel to the fingers; and, on being exposed to the flame of a candle, will yield a considerable quantity of fat. Although its absolute weight, when enlarged, may exceed that of the healthy liver, I am disposed to think that its specific gravity will, in perhaps the majority of instances, be found to be less; thus a liver apparently healthy was of the specific gravity 1·0625; whilst the specific gravity of two fatty livers, examined at the same time, was only 1·0275. I have, however, recently met with a case, in which the liver was not only much enlarged, but exceedingly dense and hard, and consequently, as I suppose, of great specific gravity."

Mr. Golding Bird, a very intelligent young chemist, analysed the bile, liver, and urine of a patient affected with this disease.

The bile was of a dirty brown colour, and contained in diffusion numerous granules of carbon. Many globules of oil appeared, after repose, upon its surface. After the addition of any acid, the most intolerable odour was evolved. The composition of the bile was:

| | |
|---|---|
| Biliary matter with an appreciable quantity of very soft fat | 6·0 |
| Earthy salts (phosphates of lime and magnesia) | 0·2 |
| Osmazome, lactate of soda and common salt | 1·0 |
| Water | 92·8 |
| | 100. |

The liver was next examined, for the purpose of ascertaining the quantity of fat present. One thousand grains were cut into very small pieces, and boiled in repeated portions of alcohol; the solutions being filtered, whilst nearly boiling hot, through muslin. By this process, a solution of fat and osmazome, with saline matter, was obtained: the fluid was evaporated, over a vapour bath, to dryness; and the residue digested in boiling water, to remove the salt and osmazome. The insoluble portion consisted of a soft brownish fat, very fusible, and possessing a peculiar and unpleasant odour: the quantity yielded by the one thousand grains was 47·6 grains, equal to 333·2 grains in each pound of the liver.

The urine was of very low specific gravity, only 1·007—quite neutral—held mucous flocculi in suspension—yielded a precipitate on protracted ebullition, which differed from albumen in not being precipitated by nitric acid—contained minute traces of the earthy phosphates—and left, after evaporation to dryness, a residue of a peculiarly bitter taste, not owing to the presence of bile.

The following appears to be the principal symptom on which Dr. Addison leans in diagnosis. It is a peculiar expression of the countenance, or rather a peculiar texture and aspect of the integuments. When strongly marked, it is indicative, if not pathognomonic, of the malady.

" It is purely integumental; as it is not confined to the face, but may pervade the whole surface of the body; although I am disposed to think that it is earliest observable, as well as most conspicuous, in the integuments of the face and backs of the hands. To the eye, the skin presents a bloodless, almost semi-transparent and waxy appearance: when this is associated with mere pallor, it is not very unlike fine polished ivory; but when combined with a more sallow tinge, as is now and then the case, it more resembles a common wax model. To the touch, the general integuments, for the most part, feel smooth, loose, and often flabby; whilst, in some well-marked cases, all its natural asperities would appear to be obliterated, and it becomes so exquisitely smooth and soft as to convey a sensation resembling that experienced on handling a piece of the softest satin. Whether this condition of the integuments precede or follow that of the liver, and whether the two are necessarily associated in every instance, I am by no means prepared to offer a decided opinion; but having, on several occasions, confidently and correctly predicted finding the fatty liver, from having observed the particular condition of the integuments just described, I have ventured to make the crude fact known to the profession, in the hope, that, attention being once directed to such a starting point, something more interesting, and of greater practical utility, may result from its further investigation."

M. Louis, it is well known, has dwelt on the frequent co-existence of a fat liver with phthisis, and even estimates, that it occurs in one-third of the whole number of phthisical patients in France; as in 120 cases, he met with 44 instances of the complication; and he even goes so far as to regard it as a mere part or consequence of such tubercular disease. He appears to have been led to this conclusion, by observing, that in almost every case of fatty liver there existed more or less indication of tubercular disease in the lungs; and that the development of the fatty degeneration of the liver is not materially influenced either by the age, sex, or constitution of the patient; except, inasmuch as these circumstances affect the phthisical disease, upon which he considers it to depend. All who are much accustomed to post-mortem examinations are familiar with the fatty looking liver so frequently found in the bodies of the phthisical. Whether this and the fatty liver described by Dr. Addison, which is attended by the peculiar condition of the integuments, and which may and does exist as a disease per se, are really the same organic alteration, or whether there are differences between them which have hitherto eluded observation, are questions which, for the present, must remain undecided. Dr. Addison will, no doubt, prosecute the inquiry, and the publication of his remarks will induce other physicians to do the same.

That fatty degeneration of the liver is not necessarily accompanied by tubercular disease, either in the lungs or elsewhere, is certain. Three such cases have occurred within the last two months under Dr. Addison's own observation. In two of the cases, no suspicion of disease of the lungs or of the liver had been entertained up to the period of the death of the patients. One of them, aged 32, died of organic disease of the brain—a second, aged 57, of pericarditis, succeeding an attempt at suicide. In another, we believe the third case, the state of the liver was predicted during life, from the condition of the integuments. In this case there was anasarca of the feet and loins prior to death, and it is doubtful whether this alteration of the liver may not give rise to dropsical effusions.

On the causes, the progress, and the results of the disorder, we possess no positive information. The conjectures of Dr. Addison upon these points may be cited, because they serve to exhibit what we do *not* know, and what we must therefore endeavour to learn. We shall cite them, and we trust that, ere long, the industry and the ingenuity of this talented physician will fill the hiatus, which his candour now points out.

"With respect to the causes of this fatty degeneration of the liver, very little, or absolutely nothing, is known. In most of the cases which I have met with, there has been either positive or strong presumptive evidence that the individuals had indulged in spirit-drinking; and indeed the most exquisite case I ever saw in a young subject, occurred in a female who had for some time subsisted almost exclusively on ardent spirits. On the other hand, the extreme frequency of the degeneration in France, where the people are but little given to such indulgences, throws considerable doubt upon such an origin of the complaint; whilst its great frequency there, in conjunction with phthisis, would almost lead to a belief that it has some connexion with a scrofulous tendency—

a belief which, I confess, I am strongly disposed to entertain.

Connected with scrofula or not, it remains to be ascertained, whether it be in reality an original disease of the liver: or whether it may not, in every instance, be merely secondary to some other or remote disorder, is not yet determined. Future experience, also, must discover what are its immediate, and what are its remote consequences; —whether feebleness of the powers generally, and of the digestive organs in particular, be sooner or later induced by it, in every instance where patients escape other mortal diseases;—whether, by impairing the powers of the constitution, it may not, in some instances, prove rather a cause than a consequence of phthisis;—whether anasarca, or any other form of dropsy, be peculiar to certain stages or degrees of it;—and, lastly, what influence it exerts upon the brain and nervous system generally, and consequently upon the feelings and disposition of the individual."

### II. Observations on Jaundice, more particularly on that Form of the Disease, which accompanies the diffused Inflammation of the Substance of the Liver. By Dr. Bright.

Dr. Bright proposes the following classification of the causes of jaundice:—

1. Congestion of blood in the liver. 2. Obstruction of bile in the biliary ducts, and more particularly in the larger ducts. 3. Chronic change in the structure of the liver. 4. Inflammatory action of the liver.

1. Congestion of blood takes place in the liver under various circumstances, of which the following are the most frequent:—Obstruction to the circulation in the chest, more particularly from valvular disease, or dilatation of the heart; constipation; pregnancy; and certain conditions of the circulation, connected with remittent and other fevers.

2. Obstruction to the passage of the bile in the larger ducts takes place from biliary concretions; from malignant or other growths in the liver itself; or in glands about Glisson's capsule; from changes in the coats of the ducts themselves; from inflammation of the duodenum; or from induration of the pancreas.

3. The chronic changes in the structure of the liver are of various kinds; sometimes the result of simple inflammatory action of a somewhat acute character, imperfectly subdued; at other times, the result of a very slow and chronic action, affecting either the secreting portion or the cellular tissue; —at others, the result of degeneration, or of malignant action taking place extensively throughout the organ.

4. The inflammatory action of the liver, which chiefly gives rise to jaundice, occurs in its substance; sometimes, if unchecked, going on to suppuration; but at other times producing a gradual change in the texture of the liver.

The gist of Dr. Bright's observations may be found in his account of the jaundice which arises from inflammatory action in the substance of the liver. His remarks on the previous forms are judicious, but need not be methodically noticed. There are two or three points to which we shall advert. They are these:—

*a.* In referring to the jaundice dependent on obstruction to the biliary ducts, Dr. Bright observes that the evacuations from the bowels, accompanying the complete retention of the bile, and occurring in cases where pressure is made on the common and perhaps on the pancreatic duct, are not undeserving of attention.

" I refer to the evacuation of fatty matter, more or less mingled with the fæces. In several cases where the obstruction has been in the pancreas itself, a considerable quantity of that substance has been seen floating, and completely separated from the fæces; of which another instance, besides those I have already published, has lately occurred to me in the hospital. But in most cases of very obstinate jaundice, accompanied by complete obstruction, an unusual quantity of fat has been detected; and my friend, Mr. G. O.

Rees, has kindly undertaken several analyses for me, with a view of ascertaining this fact; the results of which may, at some future time, be more fully stated."

*b.* Organic alteration of the liver is well known to be sometimes attended with diminution, not augmentation, of its bulk. Dr. Bright's observations on the structural condition of the organ may be quoted.

"The liver is sometimes increased in its size; but very frequently quite the contrary, the organ having evidently undergone contraction in the progress of the disease; indeed, I have, in some cases, most distinctly traced its enlargement in the beginning of the attack, and its gradual diminution towards the more-confirmed stages of disorganization. Though the larger ducts are pervious, and a certain quantity of imperfect bile is found in the gall-bladder, yet the whole substance of the liver is frequently tinged by the bile retained within its smaller ducts. A general granular appearance exists throughout the liver, as if the acini were drawn into masses, surrounded by thickened cellular membrane: and if, without being injected, a portion of liver in this state is macerated in water for several weeks, the little granules assume the appearance of adipocire; and may be easily washed out by a stream of water, leaving a fine tissue of vessels and cellular membrane floating in the water. When the disease has gone further, the bands of cellular membrane are seen intersecting the structure, and forming more decided septa between the masses of acini. These are the more common appearances, where, from frequent over-stimulation, gradual change in the structure of the liver has given rise to jaundice."

*c.* Where the structure of the liver has undergone some chronic alteration, ulceration of the lining membrane of the colon is a common consequence. In no case is it more frequent than when the true fatty degeneration, which is seldom accompanied by jaundice, has occurred.

We may now proceed to that form of jaundice which depends on a state of inflammatory action, more or less generally pervading the substance of the liver. Dr. Bright remarks that it is highly probable that in different cases different constituent portions of the liver are particularly implicated—a probable conjecture, that implies our yet imperfect acquaintance with the affection. The causes from which this inflammatory jaundice arises are frequently very different, and, besides irregularities of diet, include atmospheric exposure, the effects of external violence and injuries, the irritating effects of biliary concretions and of the retained bile, and perhaps the stimulating action of mercury. The symptoms likewise differ in many respects from those of the other forms, though not separated by sharp and abrupt lines of demarcation; and the changes produced on the substance of the liver are decidedly different, affecting much more generally the secreting portion than the connecting cellular tissue, and probably involving the branches of the portal vein in preference to other parts.

Dr. Bright divides the cases into acute and chronic. We cannot materially abridge his history of the disease, which we shall give with some slight curtailment in his own words.

"It frequently comes on very insidiously, with symptoms and feelings of general constitutional derangement, depression of spirits, slow pulse, oppressed breathing, wandering abdominal pains, constipated bowels, and sometimes sickness of the stomach. In a day or two, the conjunctiva becomes tinged; and in a few days more, there is universal bright bilious suffusion of the skin. It is now found that the pulse is either accelerated, or sometimes still oppressed; and frequently, on pretty severe pressure about the region of the liver, some degree of tenderness is manifested; while in other cases, pressure produces little or no immediate suffering, but the pain comes on gradually a short time after the pressure has been made, and continues for hours or days. Cases of the less acute kind generally yield readily to treatment, if it be adopted early; and they form a large proportion of the cases of simple jaundice which present themselves in practice. In other cases, the inflammatory action is attended with much more severe symptoms, with considerable pyrexia, quick pulse, flushed countenance, and dry tongue, while a jaundice of the most intense colour is diffused over the whole surface. The stools are, both in the more and less acute cases, of a light colour; but less decidedly so, and subject to greater variations than when the obstruction is mechanical; and occasionally, after a few days, give little evidence of deficiency of bile. The urine is deeply tinged. When the disease assumes its more active and febrile form, those symptoms referrible to the brain and nervous system, and which appear to depend upon the deleterious effects of bile circulating in the blood, are more intensely marked than in any other form of jaundice; and the tendency to hæmorrhage sometimes comes on very early, and is excessive. In some cases, rigors, which assume the form of irregular intermittent paroxysms, form a prominent feature, as the disease advances; and then it often happens, though not always, that suppuration is established; and this may be going on to a great extent, while still the jaundice has rather decreased, or varied exceedingly in its intensity."

The changes which occur in the structure of the liver vary with the period at which the disease has terminated fatally. In general the size of the organ is not materially increased, but not unfrequently perceptibly diminished. There is no accumulation of bile in the minute ducts, and the yellow tinge of the liver is not considerable. The gall-bladder contains little bile, and its mucus is sometimes scarcely coloured by it.

When the disease has terminated early in its course, the whole liver feels rather soft and flaccid; the surface appears variegated, of a light-yellow, and dark-red or purple, in patches; and certain portions project above the rest, which, when cut through, sometimes prove of a softer texture, and even to be undergoing a process of change or disorganization; and portions of the same kind are intermixed throughout the whole substance of the liver; while, at other times, the yellow portions are harder than the surrounding substance.

When the disease has not terminated early, and the colour of the skin has subsided to a light lemon yellow, the structure of the liver is found extensively altered, and a great many of the acini are apparently incapable of receiving their due quantity of blood. They are then of a whitish-yellow colour, and rather hard and contracted, than enlarged; and these altered acini are seen in groups and clusters, which, on careful examination, will generally be found to follow the course of the divisions of the portal vessels, so as to be disposed around them like a sheath, which sometimes extends to the thickness of a quarter of an inch.

In other cases, this diffused inflammation of the substance of the liver ends in suppuration. We find a multiplicity of abscesses, all of which seem tending to discharge themselves into branches of the portal vein, which then assumes a most unhealthy suppurative appearance along large portions of its course.

Dr. Bright's suggestions with regard to treatment are such as would probably present themselves to the mind of a well-informed practitioner. Yet they may be usefully extracted. That treatment must, of course, be essentially antiphlogistic—general bleeding in the more violent forms—cupping and the application of poultices in the less severe ones.

"The combination of calomel, antimony, and opium, must be occasionally administered; and antimonials must be combined with the purgatives, which, in the form of pills, should be given to act regularly on the bowels, and should be aided occasionally by the sulphate of magnesia and other saline purgatives: and, in many cases, the saline purgatives, alternated occasionally with mercurials, are sufficient to cure the disease. A free and uninterrupted action from the skin is most desirable; and to promote this more effectually, the warm-bath may be very advantageously employed; and the poultice, while it restrains the patient in bed, assists forcibly, as a diaphoretic measure.

Under treatment of this kind, a very large proportion of cases are completely cured: and where the result is otherwise, it generally arises from some complication of diseases; most frequently from previous disorganization of the liver, or from neglect, on the part of the patient, in not applying for medical aid, or in not steadily pursuing the plan laid down: for it is not uncommon to find persons inclined to make light of an attack of jaundice, if the pain or inconvenience attending it is not such as to prevent them altogether from pursuing their usual occupations. Nothing, however, can be more injudicious; and it is the duty of the practitioner to impress this upon his patient: for it can never be a matter of slight importance, that either obstruction or inflammatory action should exist in the intimate structure of a secreting organ, still less in an organ so delicately complex as the liver: and there is no reason to doubt, that although, even without treatment, an attack of this kind may pass off, the liver will be some time before it has completely recovered; and thus a tendency to relapse, or to a renewal of the complaint, may occasionally be observed, shewing itself at longer or shorter intervals throughout life, and terminating, at length, in the destruction of all the powers of the stomach, the ulceration of the colon, miserable emaciation, or universal dropsy."

Eight cases follow, and occupy twenty pages of the Report. It is unnecessary, indeed it would be impossible, for us to detail these. A sample of a slight case, and of a severe one, will be sufficient to illustrate the preceding observations.

Case 1. *Jaundice following Intemperance and Exposure.—Cure.*

W. B. æt. 18, admitted Nov. 30, 1831, with jaundice of a clear bright tinge. He had been a hackney coachman for five years, and had been addicted to porter and spirit drinking, and to gonorrhœas. Six weeks before his admission his mouth had been made sore by mercury—seven days before admission, he found his eyes yellow, and jaundice followed. The abdomen was tense and hard—the tongue white and coated—the bowels not open since the preceding day—pulse 82, full, but sluggish. He complained of pain in the right side of the abdomen, which he said had been coming on for three months.

Habeat Pilul. Colocynth. c̄ Cal. gr. xij. statim; et Haustum Sennæ vespere, si opus fuerit.

Mistura effervescens.

*Dec.* 1. Jaundice more general and deep. Two copious stools, solid, and of a dark clay colour: urine of a deep colour, like the infusion of senna: pulse 81, full and jerking: tongue cleaner.

Habeat Hydrarg. Submur. gr. i. sextis horis.

The calomel was repeated in these doses for ten days; after which blue pill was given, at longer intervals; and during the whole time infusion of senna and magnesia, and sulphate of magnesia, were repeated till the bowels were freely opened. After the lapse of one month, he was discharged, quite well.

The next case was that of a free drinker, with nearly similar symptoms. The jaundice had followed exposure to cold. Cupping, calomel, taraxacum, and subsequently blue pill set the patient to rights.

The remaining six cases were all fatal, and dissection rendered them complete. In two suppuration occurred. It may, perhaps, admit of question whether it is so clear as might be wished, that inflammatory action was in all the cause of the symptoms, indeed the distinction between jaundice depending on congestion of the liver, and that which arises from inflammation of its structure, though evident on paper, is not always so precise in practice. In the fourth case of the present paper, the evidence of inflammation in the liver is by no means satisfactory.

We shall present an abstract of the fifth case, because it was one which occurred early to our author, and first drew his attention particularly to the subject.

CASE. *Jaundice, without Mechanical Obstruction—Fatal.*

Mrs. S. aged 28, a drunkard, was admitted Aug. 6, with suspicious sores, for which she was ordered Plummer's pill and sarsaparilla. On the 13th Nov. she was turned over to Dr. Bright. She had had pain in the abdomen for a week, and had been jaundiced for two days—the bowels were rather confined—the pulse was moderate, but quick. *Cupping—Hyd. c̄. Cretâ—Ol. Ric. followed by leeches.*

The yellowness increased—the abdominal tenderness continued—the stools remained clayey. The pulse was about 96—there was thirst—occasional sickness. Cupping, leeches, mercurial purges, blue pill, and fomentations constituted the treatment.

On the 1st of December, her pupils were rather dilated, her utterance was indistinct, and there was loss of power in the left hand. On the 2nd, she was drowsy—in the evening she was screaming loudly—and on the 3rd she was quite comatose. On the 4th she died.

*Dissection.* The whole body was intensely yellow.

The whole of the vessels and sinuses of the brain were unusually loaded with blood—on slicing the brain many bloody points made their appearance—the ventricles were unnaturally dry—the arachnoïd serum was yellow.

The liver weighed only two pounds five ounces. It was soft or flaccid to the touch; quite free from any mark of peritoneal inflammation. Its external appearance was mottled dark-red liver-colour, with yellow stone-colour. The acini were pretty distinctly to be traced throughout—red at their centres, and yellow in their circumferences; and in most parts the yellow bore a large proportion to the whole. The gall-bladder was contracted; and contained about half a drachm of mucus, very slightly tinged with green. The ducts were all pervious and healthy, and were not even stained with bile.

As there was no obstruction to the passage of the bile, and as there was not any trace of it in the liver, it must have been absorbed into the system almost as soon as it was formed. Dr. Bright "is inclined to consider this as an example of a decidedly inflammatory state of the organ." It may be so, and yet it is difficult to say where the positive evidence lies. The symptoms and organic lesions tend to support the idea of inflammatory action, but neither can be said to prove it satisfactorily. On the other hand, the peculiarity of the hepatic circulation must be taken into the account, and will induce us to demand proofs of vascular action, somewhat different from those obtained in organs freely supplied with arterial blood. On the whole, we may conclude that the subject deserves investigation, and that further researches may reasonably be expected to clear up what is yet obscure, and to determine what is still indefinite.

We shall terminate this notice with a case in which suppuration took place in the substance of the liver.

CASE. *Slight Jaundice — Numerous Abscesses in the Liver.*

S. T. aged 47, admitted Jan. 6, 1836. Two months previously, having suffered for a short time from pain in his right side, he was exposed to damp and cold. A rigor was the consequence. The rigors returned, and the pain in the side increased. At the time of his admission they generally came on at the hours of eleven in the forenoon, and four in the afternoon; and were generally preceded by a violent cough, unattended by expectoration. He likewise, frequently, had a rigor when he awoke out of his sleep. He had never been completely jaundiced; but had now a very sallow complexion, with a decided yellow tinge; and the conjunctiva tinged with bile, while the countenance was peculiarly anxious and dejected: tongue covered with a yellowish-brown fur in the centre, and a whitish fur at the edges. He complained of pain in the upper part of the abdomen, and also in the loins. The slightest pressure on the region of the liver gave him considerable pain: pulse irregularly intermittent, and jerking: some headache. There seemed scarcely any room to doubt that the liver was, in this case, the seat of inflammatory disease.

Cupping, blue pill, calomel, and carbonate of soda in full doses constituted the treatment.

Occasional rigors—slight jaundice—pain, sometimes greater, sometimes less, in the right side—a pulse quick, sharp, or full, and intermittent—a dry tongue—and a warm skin, composed the principal symptoms. On the 13th the patient died.

*Dissection.* "The skin was universally jaundiced, of a light colour: body by no means emaciated. The lung on the right side was pushed up by the liver, which rose as high as the fourth rib; but the lung itself was perfectly healthy. The liver was decidedly large; and adhered pretty generally to the parietes, and, on its concave surface, to the different viscera on which it rested. Some parts of the substance of the liver appeared healthy; but the whole of the right lobe was pervaded by numerous abscesses; so much so, that a section through the thick part divided at least thirty, chiefly filled with yellow and tolerably healthy pus. Many of them were contained in cysts not unlike, in texture, the parietes of a suppurating tubercle in the lungs. At the thin margin of the right lobe, one of these abscesses shewed itself externally, presenting an irregular gangrenous patch, of the size of a half-crown, and, when cut into, proved a sloughing abscess: but the peculiarity of all this extensive suppuration was, that it seemed to implicate most generally the system of the portal vein; for throughout we cut into large trunks of this vessel filled with pus, and apparently in free communication with the abscesses, both large and small; so that it was very difficult to say, from the inspection of the parts, whether the mischief began in the veins, or was communicated to them. The veins themselves, however, were in a state of suppuration: they were thickened, and their surfaces appeared imbued with suppuration, like the walls of one of the abscesses. The veins going to the cava were quite healthy, and contained blood of natural appearance. The large ducts of the liver were also healthy; and the gall-bladder contained a full ounce of light-coloured bile. The diaphragm, where it had been in contact with the liver, was beginning to be implicated in the suppuration; and the mark was visible through its whole thickness, discolouring the pleura."

### Case of Cystine or Cystic Oxide Calculus, with Remarks on the existence of Cystine in Urinary Deposits. By Mr. Golding Bird.*

Cystic oxide is not a frequent constituent of urinary calculi. It was discovered in 1810 by Dr. Wollaston, and most of what we know of it is due to that physician, and to Drs. Marcet and Prout. Mr. Bird is led to think that cystine is more common, both as a constituent of calculi and of urinary deposits, than has been supposed.

On the physical and chemical characters of cystine and of cystic calculi we need not dwell. We refer those who are ignorant of them to the work of Dr. Prout. But we may observe that although the cut surfaces of nearly all the cystic calculi that have been described, have exhibited a blueish green tint, Mr. Bird had lately an opportunity of examining a calculus of this kind, whose section was of a yellowish-brown tint, bearing no distant resemblance to the colour of the fawn-coloured urate of ammonia calculi.

In the museum of Guy's Hospital, are ten specimens of cystic oxide calculi. Four have been described by Dr. Marcet; six have been placed in the museum subsequently. One of these calculi is remarkable for its singular shape, in which it exactly resembles a lady's ear-drop, having probably assumed this form during its residence in the pelvis of the kidney: it is about an inch long, and, like the others, possesses a pale-green colour: indeed, from the frequent occurrence of this tint in calculi composed of cystine, it has been looked upon as being quite characteristic of its presence; and this circumstance, Mr. B. is inclined to think, has been the cause of these calculi, when

* Guy's Hospital Reports.

not possessing this colour, being occasionally overlooked in large collections.

A calculus composed entirely of cystine has lately been removed from the urethra of a boy by Mr. Key, and as the particulars of such cases are rare, and it is desirable to ascertain the circumstances under which cystic oxide calculi are formed, the case deserves to be recorded. It is related in Mr. Key's words.

"The subject of the cystic oxide calculus was a young gentleman, aged about 12 years, of a delicate complexion, with hair and eyes inclined to auburn, and in his frame exhibiting general delicacy of texture. His parents, though healthy, are not strong persons, but have not been disposed either to calculous disorders or to gout. The prevailing disposition in the family is a tendency to strumous affection; under which, in a somewhat aggravated form, a younger member of the family is now labouring. The boy from whom the stone was taken had never, as far as his mother could recollect, shewed signs or expressed symptoms of kidney-affection; nor had his urine been noticed as peculiar. The only circumstance that his parents had remarked, was, that he could not eat the meat at school, on account of its being less nicely dressed than that to which he had been accustomed at home; and his diet, therefore, had been less nutritious than usual. The attack which brought the stone to light was sudden. On the afternoon of the 24th of May 1836, he complained of difficulty and pain in voiding his urine; and in the evening he was able to void only a few drops at a time, and at frequent intervals. About midnight, the urine ceased to flow, even after violent efforts to pass it; and I was requested to see him. On attempting to introduce a catheter, I was checked by a foreign body lodged in the urethra, just anterior to the scrotum, which entirely occupied the canal of the urethra, and seemed to be firmly impacted. From its size, I judged it to be too large to escape by the glans; and therefore extracted it by an incision through the corpus spongiosum. It proved to be a rather large calculus, of an oval shape, and studded with minute tubercles: its colour struck me as being peculiar: it had not the nut-brown or reddish colour of the lithic acid, but the amber tint of the cystic oxide formation, and also the translucent appearance characteristic of that species of calculus. The wound has healed quickly; and his health remains good. He is passing the Summer at the sea-side."

The calculus presented, on making a longitudinal section of it, the same confused crystalline structure as the other specimens of this species of calculus, but did not possess any of the green colour usually present; its tint being exactly that of the fawn-coloured urate of ammonia caculus: its specific gravity was 1·777. We need not mention its behaviour to chemical re-agents. That proved that it was cystine.

"I could not detect," says Mr. Bird, "the presence of muriate of ammonia in this calculus; although I have discovered this salt in almost every other species, having always looked for it since reading Dr. Yellowly's paper upon this subject in the Philosophical Transactions. As the boy, from whom this calculus was removed was under treatment when I received it for examination, I was exceedingly anxious to obtain specimens of his urine, to ascertain whether it deposited any thing, and if so, what this deposit consisted of. On mentioning this to Mr. Key, he very kindly procured for me, at different intervals, specimens of the urine; in which I very readily detected cystine, not only as a deposit (as I had anticipated), but also in solution. This urine was pale, of low specific gravity, generally being about 1·0148, without any action on litmus or turmeric papers; and contained, in diffusion, clouds of mucus, mixed with a white, crystalline deposit, which readily subsided to the bottom of the vessel, and closely resembled a deposit of the ammoniaco-magnesian phosphate; from which it was readily distinguished by a microscopic examination, presenting the appearance of flat six-sided tables, which I have already mentioned as quite characteristic of cystic oxide. On applying a boiling temperature to the urine, after the deposit had been diffused by agitation, it did not become clear, until after the addition of a considerable quantity of muriatic acid: acetic acid did not, under similar circumstances, dissolve it; as it would have done had the deposit consisted of earthy phosphates or oxalates. Ammonia, with the aid of heat, readily dissolved this deposit.

On submitting this urine to quantitative analysis, after being freed from the deposit by filtration, I found it to consist of,

| | |
|---|---|
| Water . . . . . . . . . | 974.444 |
| Urea, with alkaline chlorides, phosphates, and lactates . | 5.7 |
| Aqueous extract, with alkaline sulphates . . . . . . . | 14.7 |
| Albuminous matter and earthy phosphates . . . . . | 4.8 |
| Uric acid and adherent mucus | 0.016 |
| Cystine (cystic oxide) . . . | 0.340 |
| | 1000. |

I was surprised to find cystine in *solution*, in this urine, knowing its total insolubility in water: for this circumstance I am unable to offer any very satisfactory explanation. Perhaps it might be held in solution by the excess of acid in the biphosphate of ammonia, which is a constant constituent of urine; or even by means of carbonic acid, which exists in every specimen of urine I have hitherto examined; and which certainly appears to be the solvent for the earthy phosphates, when they exist in urine in an anormally large proportion; a fact that has been, in my estimation, fully proved by the late experiments of my friend Mr. R. H. Brett (*Med. Gaz. March* 1836). I may remark, that the urine, of which the analysis is above mentioned, was passed by the patient on May 28th, being four days after the removal of the calculus. I examined different specimens of the urine passed by the same patient at different intervals; viz. June 7th, 14th, and 27th; which differed from the first specimen, in the gradual decrease of albuminous matter and cystic oxide, and increase of urea: indeed, on that passed in June 27th, not a particle of cystic oxide could be detected: but whether this circumstance indicates the removal of the diathesis, or the formation of another concretion, must be left to time to determine."

Mr. Bird concludes by adverting to an opinion of Dr. Marcet and of Dr. Prout, "that the cystic diathesis generally exists, to the exclusion of other diatheses; and that this diathesis does not seem readily to change into any other."

Mr. B. does not think this correct, because a section of a calculus contained in the Museum of Guy's Hospital, exhibited, on close examination, the following substances arranged in distinct and separate zones.

1. A nucleus of oxalate of lime.

2. A zone of green cystic oxide, about $\frac{1}{36}$ inch thick.

3. A zone of urate of ammonia, *mixed* with fawn-coloured cystic oxide, $\frac{1}{10}$ inch thick.

4. A zone of green cystic oxide, about $\frac{1}{20}$ inch thick, resembling No. 2.

5. A layer of the urates of ammonia and soda, $\frac{1}{16}$ inch thick.

6. Alternating layers of urates of ammonia and soda, with oxalate of lime, about $\frac{1}{10}$ inch thick.

"The section of this calculus thus acquaints us with some very interesting and important facts; that not only can the cystic oxide *replace* other diatheses, but it can exist *simultaneously* with them. Thus, when this calculus was *first* formed, the oxalate of lime diathesis must have prevailed;—that this must have been replaced by the cystic oxides;—that, subsequently, the urate ammonia diathesis must have existed for a time contemporary with that of the oxide, and then disappeared, again leaving the cystic oxide diathesis prevailing: this, in its turn, had again been replaced by the diathesis of the alkaline urates; which finally alternated with the oxalate of lime, until the calculus ceased to increase. The examination of this calculus thus effectually disproves the assertions of the two chemists above mentioned, respecting the *exclusive* nature of the cystic oxide diathesis."

We cannot perceive that this one fact at all justifies the tone of brusquerie and dogmatism adopted by Mr. Bird. His good taste may reasonably be questioned, when it is remembered that the gentlemen, whom he addresses so rudely as two chemists, have done great service to the profession by their researches on calculous disorders, and dignified it by their high scientific attainments. Had Mr. Bird been Berzelius, which he is not, he would have been esteemed ill-mannered — being Mr. Bird, he appears in a still more unfavourable light. And after all, what is the pother about? Dr. Marcet and Dr. Prout conjectured "that the cystine diathesis *generally* exists to the exclusion of other diatheses; and that this diathesis does not *seem readily* to change into any other." Nothing could be more cautious, or more philosophical than such an observation. It is the expression of the facts that were known at the time of its enunciation, and its modesty and good sense contrast strongly and pleasingly with the dogmatic contradiction of Mr. Bird—a contradiction founded on the arrangement of *one* calculus. That exception, or several others to boot, would not really contravene the original position of the "two chemists." We take notice of this, because Mr. Bird is a gentleman of much industry and ingenuity, and it were a pity to see them marred by bad manners or bad taste.

### Remarkable Malposition of the Stomach, which lay in the Thorax.

There have been at various times, cases reported, of hernia of the stomach into the chest, either through a congenital, or an accidental, or morbid opening in the diaphragm. Such cases are fortunately matters more of curiosity, than of much practical importance, yet they occur sufficiently often to render ignorance of their possibility inexcusable. We shall, therefore, present the following case.

*Case.* Sarah Charington, aged 19, was very delicate from her childhood. At three months of age she had a severe attack, which was attended with sudden shortness of breathing, and was considered an inflammation of the chest. When nine months old she had vomiting to an alarming extent; and when four years of age, suffered a most severe attack of the same kind, accompanied by purging; and the medical man who then saw her pointed out the peculiar conformation of the chest, and the little chance that the child would reach to years of maturity.—Before the age of five she had passed through the measles and the hooping-cough pretty favourably, and at eight years had a severe attack of fever. During her childhood she was always unable to play with other children, or to bear with any rough treatment; and, on several occasions, was expected to die from sudden dyspnœa. Her stomach was always liable to be deranged by the slightest cause; and she never had a figured motion till fourteen years of age. About that time, however, her bowels became costive; and in that state they ever after continued. She grew fatter and stronger, and had some colour in her cheeks: nevertheless, she enjoyed no good health. Her appetite was capricious; she could scarcely walk upright; and if she lay on her left side, it produced great uneasiness, and frequent vomiting: she was most easy when lying on her back, with her shoulders raised, and her knees bent towards her body, or sitting doubled on a chair: she had frequent palpitation, which she used to compare to the fluttering of a bird in her chest. The catamenia appeared once, at the age of seventeen; and only twice afterwards, at very long intervals; there was frequently a slight leucorrhœal discharge.

She referred the sensations of nausea to the left side of the chest and sternum, where there was a frequent gurgling noise. Kneeling was an easy position to her—exertion of any kind was next to impracticable. Her state, however, varied a good deal.

Towards the end of April 1835, she was seized in the street with violent sickness, vomiting a large quantity of brown matter; and after that time she was more subject to vomiting than before. She lost flesh, and became obviously more out of health. She was sent down to Ipswich in July, to be put under the care of Mr. Sampson, from whose advice she had formerly derived much advantage. She returned to town decidedly improved; but soon becoming worse, was admitted into Guy's Hospital, under the care of Dr. Bright, on the 14th of October, 1835. She was then emaciated, constantly vomited her food, and could not lie on the left side, because it brought the vomiting on. The pulsation of the heart was quite distinct upon the right side, but could scarcely be perceived upon the left. She said that this had always been the case. On the 7th of November, she was so much relieved that she went out, three days after which she took a long walk, when the vomiting returned, with obstinate costiveness, and pain in the back. She died on the 13th of February, 1836.

*Dissection.* The body was considerably emaciated, and the left side of the thorax had decidedly fallen in: the form of the chest was likewise remarkable, from its great length, the ribs reaching within a short distance of the crest of the ilium. On removing the sternum, the heart was seen lying in its pericardium almost on the right side of the chest, being considerably beyond the middle line; and on its left lay a tumor of a red or fleshy colour, somewhat vascular, slightly lobulated, and elastic to the feel. This tumor rose to the fourth rib; and a small portion of the lung appeared overlapping it from above. On the right side, a thin flap of the lung descended to the seventh rib, lying upon another tumor not unlike that on the left cavity of the chest. In the abdomen, the liver occupied a considerable space, extending a little more to the left side; the stomach was no where to be found; while the arch of the colon was seen passing across the abdomen, just below the liver; and an elastic tumor arose from under the acute edge of its right lobe.

On further examination, it appeared that the two bodies which encroached on the right and left cavities of the thorax were formed by the stomach contained in a membranous bag, over parts of which a scanty distribution of muscular fibres could be traced; when this bag was cut open, the stomach, covered with the omentum containing the usual deposit of fatty matter in its meshes, came into view. The œsophagus was now traced; which commencing in its natural way, terminated in the cardiac portion of the stomach, on a level with the body of the fourth dorsal vertebra.

The stomach, after filling all the lower part of the left cavity of the chest, passed behind the apex of the heart, where it was somewhat contracted; and again expanding, filled in the same way the lower portion of the right cavity, but to a less extent: the canal then passed through the diaphragm, in a situation nearly corresponding with the passage of the cava; and the duodenum running in a straight line downwards, behind the liver, was involved in a fold of peritoneum, where it was connected with the head of the pancreas, and accompanied by the cystic, hepatic, and common bile-ducts: the intestine then dipped behind the mesentery and mesocolon, and pursued very nearly their natural course.

The cyst which enveloped the stomach was a membranous muscular covering, not in any part adherent to the stomach; and appeared to be formed by a congenital splitting of the diaphragm; for on both sides we could distinctly trace bundles of muscular fibres coming off from the part where the diaphragm arises, and passing above the stomach towards the mediastinum; where they became so attenuated, and the cyst so membranous and mingled with cellular membrane, that all traces of muscular structure were lost. On the left side, there seemed to be even a third division of the diaphragm, in which was observed no muscular structure—going to envelop the spleen, and surrounding it by a membranous bag, out of which it could be turned, like the heart from the pericardium. The spleen was small, hard, and fleshy. The pancreas held its usual relation to the spleen.

The stomach was very large; and the fluid it contained nearly filled a wash-hand basin. Its internal surface was throughout of a dusky red, and its texture generally thickened; but in the part corresponding to the small curvature were found two or three deep chronic ulcers, with raised edges of almost cartilaginous hardness, one of which would have contained a small walnut in its cavity. The pylorus was very large and open, and seems to have been situated just above the diaphragm. The duodenum was greatly dilated at its origin, and partook of the appearance of the stomach.

The elastic tumor observed below the margin of the liver on the right side, proved to be the pelvis of the kidney, distended to the size of a tea-cup with clear urine; the kidney itself being much diminished by absorption.

---

### Statistics of Hospitals.

The following facts relating to medical statistics are taken from the British Medical Almanack, a notice of which has appeared in the commencement of the present Periscope. There are several subjects embraced in these generalizations, and we shall notice them as briefly as possible in succession.

1. *Do Examinations after Death diminish the number of Applicants for Admission into Hospitals?*

Most certainly they do not. The alarmists would fain terrify us with prognostications of loss of hospital patients, from the prevalence of dissections, or from the operation of the Anatomy Act. Benevolent and timid people are very apt to overrate the sensibilities of the poorer classes. No doubt their ignorance makes them liable to prejudice. But even prejudice yields to the operation of necessity, and the sense of interest. The poor may at first raise a clamour against dissection; but when their lives are at stake, when present comfort and medical attendance, with the possibility of future dissection, are balanced against distress and no medical relief, the decision is pretty surely in favour of the former. A small knowledge of human nature would lead us to anticipate this result. Experience, both on the Continent and in this country, has proved it. Indeed, the framers of our Anatomy Act might safely have taken a bolder step than they did, and the working of the bill would probably have been better than it actually is. Political considerations interfered, and still interfere, with the insertion of compulsory clauses in that Act. We say that experience has decided that the knowledge on the part of the poor, that examinations after death are the rule of a hospital, does not diminish the number of applicants for admission into that institution. Perhaps there is no hospital, in London or out of it, where examinations after death are more constantly and more systematically pursued than they are in St. George's Hospital. Yet the number of applicants far exceeds the capability of accommodation for them, although that hospital contains upwards of three hundred beds. In other hospitals the case is just the same.

"At an hospital in an agricultural district, the house surgeon for the time was allowed to examine every body—in six months 18 or twenty bodies were carefully inspected, without asking permission from the friends; this had not been the custom before, yet the average number of in-patients increased that year to 100, the admissions to 1112; the average number of in-patients the year before had been 76, the total admissions 837. The connexion of a theatre for dissection with hospitals necessarily excited more prejudice formerly, than the simple inspection of the body excites now; yet experience proved that this had no influence whatever in diminishing the number of applications for admission. Cheselden commenced courses of anatomy at St. Thomas's in 1711; in 1706 it contained 362 patients, in 1717 the number had increased to 420. For other reasons it is not desirable that theatres for dissection should be immediately connected with hospitals; but this circumstance has not at all been found to diminish the number of patients at St. Thomas's, Guy's, St. Bartholomew's, the London, or any other institution. The number of applicants for relief at an hospital depends on the *kind manner* and *reputed skill* of the officers; the poor have sagacity enough to discover that the person most zealous in investigating their diseases is best qualified to relieve their sufferings."

We may set it down as a certain fact, that neither systematic examinations after death, nor the connexion of a school of anatomy with any hospital, will in any degree curtail the amount of its patients.

A. *Numbers of Patients and of Deaths in the Hospitals of Great Britain.*

"Besides 182,847 seen at the dispensaries and as out-patients, more than 24,000 patients are treated, and 2,100 die within the walls of the London hospitals. According to the last printed reports, 30,000 are admitted, and 1780 die in the country hospitals; in the Scotch infirmaries about 6,900 are treated, and 670 die; in Ireland, it appears from official returns and Mr. Phelan's work, that 8126 are treated in the county infirmaries, 6855 in the hospitals of Dublin, and nearly 15,000 fever patients in the establishments destined for their reception. The cases of disease placed every year, in the public institutions of the country, under the observation of the medical officers, amount, therefore, to at least 90,000, 6,300 of which terminate fatally, and may be submitted to necroscopical investigation. At a moderate estimation, the dispensary and out-patients are four times as numerous. The sums expended in supporting the medical charities of the country are enormous: the value of the labour devoted in them to the treatment of the classes unable or able to pay for medical advice, can scarcely be calculated."

B. *Advantages of due Statistical Returns from Hospitals.*

It is unnecessary to dwell on the advantages that would accrue from accurate and well-digested statistical hospital reports. It is only by the combination of particular facts and of general results that much good can be done to medicine. The time has arrived when a general and a well-arranged system of hospital reporting must begin to attract serious attention. There are some suggestions in the work before us, which we will lay before our readers, and particularly before the medical officers of hospitals.

C. Dr. Clifton, who published a work intituled the State of Physic, in 1732, proposed the following plan.

"In order, therefore, to procure this valuable collection, I humbly propose, first of all, that three or four persons should be employed in the hospitals (and that without anyways interfering with the gentlemen now concerned) to set down the cases of the patients there from day to day, *candidly* and *judiciously,* without any regard to private opinions or public systems, and at the year's end publish these facts just as they are, leaving every one to make the best use he can for himself. Would not some such method as this let us more into the nature of diseases in a few years than all the books of theories, or even the books of observations, hitherto published? Certainly it would: and yet if proper encouragement were given, it is not at all unlikely, but that persons enow would soon be found, every way qualified for such an undertaking. And even if good salaries were allowed them, and every thing made as easy and agreeable to them as they could desire, the benefit the public would receive from them would vastly more than balance the expense."

There is an objection to the publication of reports from a hospital in a separate form. Such a form of publication is attended with much trouble and some expense, and, after all, as it costs money, will not be very widely circulated. The best mode of rendering the facts that occur in hospitals generally available and useful to the profession, would be their regular and periodical publication in the Journals. We would suggest the combination of reports at short and at long intervals—in a weekly and in a quarterly journal. We should have no objection to open our pages to a well-conducted system of hospital-reporting. Sooner or later, some plan of the sort must be adopted.

The object, however, at the present moment is to agree on what is the best plan of *statistical* reporting. This is not so obvious as at first sight it might appear, and the Editor of the Almanack makes some observations, which are well worthy of attention.

"The first step in medical statistics, after having determined the mortality, is to ascertain the number of attacks of sickness, at different ages, to which a population is liable, and the numbers constantly ill. Hospitals throw no direct light on these questions, or on the absolute mortality and duration of cases. But hospital statistics may show the mortality and expected duration of different diseases at several stages of their progress, and the extent to which their mortality and duration are diminished by remedial means. To make the series of observations perfect, the cases should be traced from their origin to the termination; but patients only enter the hospital at a certain period of their complaint, on the 10th, the 20th, or the 30th day, &c.; and before this time, out of 1000 attacked, 500 perhaps have either recovered or died: you then can only have to deal at the hospitals with the remaining 500, whose history you may retrace, and then follow for a mean term of thirty, forty, or sixty days; when a certain number still remain ill, but the majority are discharged cured, relieved, or dead. Because hospital cases only come under notice after a certain time has elapsed, and are not all followed to the end, it has been thought they afford little instruction. We have reason to entertain a different opinion: at the same time, let it be constantly borne in mind that the mortality is merely the mortality for certain periods of disease: from the twentieth to the fifty-sixth day, for instance—it is not the entire mortality of the class of cases. To represent this, all the deaths that occur after leaving the institutions, should be added; thus augmenting the mortality, which is however lowered, on the other hand, by another powerful cause. Admit that 1000 labourers are attacked by disease; all repair to the infirmaries, who remain ill, on an average, twenty days; but before that time, 280 have recovered, and twenty died: in the institutions 700 remain under treatment till the fifteenth day, and during this period fifty die, and 450 recover; 200 leave uncured, and of these, ten die. The mortality of the cases would be 8 per cent.; the mortality of the cases, while in the institution, was fifty in 700, or 7 per cent. It may happen that the mortality in the two instances coincides by a sort of mutual compensation between the excess of previous recoveries and the subsequent deaths. Nearly the same reasoning applies to the duration of cases."

It cannot be expected that the deaths which occur after dismissal from the institutions can be reported on. The day of the disease on admission—the occurrences whilst in hospital—and the day of discharge or of death, will probably constitute the main statistical facts that we can obtain.

D. It would be advantageous if the annual reports from all hospitals referred to the same period—from the 1st of January to the 31st of December. As it is, the reports of some hospitals start from Lady-day, or Midsummer, and thus accurate comparison with other hospitals is interfered with.

2. "*The number treated yearly is the mean of the numbers admitted and discharged.* The total number of in-patients in the year is generally published in this manner:—

In-patients admitted from Jan. 1st, 1833, to Jan. 1st, 1834.

| | |
|---|---|
| Remaining Jan. 1st, 1833.... | 46 |
| Admitted since ............... | 342 |
| Total .. | 388 |
| Discharged cured ............ | 200 |
| Relieved, &c. &c. &c. .... ... | 142 |
| Remaining Jan. 1st, 1834 .... | 46 |
| Total.. | 388 |

In some reports it seems to be implied that 388 patients have actually been treated in the year. This is an error: you have already counted the forty-six

*once* in the Report of 1832; and of the 342 admitted, you will in the same way count forty-six a *second* time in the year 1834. The forty-six patients in the house at the beginning of the year have, on an average, remained half their time—for instance, twenty days;—they will probably still remain twenty days; they may therefore be regarded as half-treated, the same as half the number fully treated; add, then, 23 to 342, and you obtain 365; but 46 remain at the end of the year only half-treated; and you have already counted them for fully treated among the 342; as a correction, therefore, deduct 23 from 365, and the original number 342 remains. 342 patients have been admitted, and 342 have been discharged in the year: this is therefore, the number annually treated. The number treated may be deduced from the number admitted and remaining, or from the number discharged and remaining: and generally, when several years are taken together, and the size of the hospital remains the same, the number discharged or admitted, exclusive of the number remaining, represents the number treated with sufficient accuracy."

### 3. *Exaggerations of Numbers of Patients.*

Hospital annalists are as anxious to multiply the number of their patients, as doctors are in private practice. Thus at some hospitals, a patient is re-counted whenever he changes his condition from that of out-patient to that of in-patient, or the contrary—or he is obliged to take out a fresh ticket every two months, and is reckoned as a fresh patient, " an ingenious zootomical device," and well calculated to knock statistics on the head. All this is, in a scientific point of view, reprehensible.

4. Patients are reported as " cured, relieved, uncured, or dead." The term cured should be employed in an uniform and rigorous sense. It is suggested that another head should be generally added—" discharged for non-attendance," a discharge which should take place, if nothing is heard of the patient for a fortnight. These non-attending patients are very convenient to reporters, and serve for cured in a wholesale way.

5. " *The annual mean number of in-patients under treatment may be determined by counting the patients every day in the year at the same hour—for instance, twelve to one o'clock—and dividing the sum by* 365: the average number of dietaries, or of persons *sleeping* in the house, would furnish the same results. When patients are admitted and discharged on a particular day, the patients discharged should be counted, if the admission and discharge take place after dinner; the patients admitted, if before: both should never be reckoned. Approximation to the average number treated may be obtained, by adding together the number in the house on a fixed day of every week or month, and dividing the sum by fifty-two or twelve. The average number of patients under treatment is exaggerated by counting the *beds* actual, or possible; by selecting the greatest number ever in the house; or by taking one day in the week, and counting, in addition to the patients then resident, all who *have been* in the house during the six preceding days. When the absentees are duly erased from the books, the average number of out-patients can be calculated in the same manner as the in-patients; the mean number that *attend* should also be ascertained. *None of the hospitals in the metropolis*, except the *London Hospital*, publish the average number of patients under treatment: but this essential statistical element is published by the Cambridge, Winchester, York, Gloucester, Exeter, Nottingham, Salisbury, Salop, Chester, Canterbury, Bedford, and Colchester Infirmaries. It is the main feature of a report, and alone controls the expenditure. It is either suppressed from imperfect knowledge or inattention; to allow the extent of the institution to be exaggerated; or to prevent the inconveniences of a check on the financial operations of the persons who distribute the funds."

The editor proceeds to detail the results deducible from the present Reports:

6. " Dividing the total deaths by the

8. The average number under treatment (5) multiplied by 365¼, and divided by the total number treated, (2) presents the mean term of treatment in days. Example: The Canterbury observations as above. The average number under treatment, 54.6, multiplied by 365¼, is equal to 199,426 days of treatment, which divided by 4254, the number treated, shows that the mean term of treatment was 47 days.

In the present hospital-books, the age, sex, residence, and occupation are generally entered. The remarkable results presented by the mortality of attacks at different ages have been pointed out in other parts of this Almanack. The sexes and occupations, as well as the ages, of the patients admitted, cured, and dying, should be inserted in the reports.

From the numbers *admitted* at each age,—for instance, 20—30, and 40—50, and enumerations of the numbers *under treatment* at the same intervals of age, it may be ascertained to what extent the term of treatment varies with the age of the patients. If for 100 treated in the year, between the ages 20—30, ten remained constantly under treatment, and for 100 admitted between the ages 40—50 fifteen remained on an average, it would follow that the patients aged 20—30 remained 36½ days, the patients aged 40—50 half as long again, or 54¾ days. The ages of the patients admitted in quinquennial periods of life should therefore be published; and, in addition to this, the mean number at the same ages constantly under treatment deduced from twelve annual enumerations made on the first day of every month."

From a review of the preceding observations it is obvious that the principal addition to reports of cases recommended by the Editor, is the stating with accuracy the duration of the disease—the number of days that the patient has been ill before admission—the number of days that he continues under treatment. Accuracy is above all things to be desired—and next to accuracy comes method. It is clear that the time is arriving when the medical officers of hospitals will find their best interest, in rendering the facts which occur in those institutions as extensively useful as possible to the profession. System will enable them to offer those facts in the best form at the least trouble. It is on this account that we have made the preceding extracts from the useful little work which lies before us. Our readers know how much we have advocated the publication of statistical results—how unceasingly we have encouraged the study and the diffusion of facts both in the form of particulars and of generalizations. We would fain hope that our endeavours "to guide and to improve the public taste" have not been altogether useless, and that our influence, temperate and rational in its objects and its character, is more permanent and more beneficial than that of mere political brawlers.

---

### Aberdeen Lunatic Asylum.

Dr. Macrobin, the zealous physician to this establishment, has published a brief report of the cases admitted and treated, between the 1st of May, 1835, and the 1st of May, 1836. There are in it some facts of a statistical character, which are adapted for the perusal of our readers. The following are the numerical statements of the patients admitted, treated, cured, or dead in the period above-mentioned.

| | |
|---|---|
| Number of Patients in the Asylum, 1st May, 1835 (date of last Report).. | 120 |
| „ Admitted between 1st May, 1835, and 1st May, 1836 .......... | 37 |
| | 157 |

| | |
|---|---|
| Whereof were considered curable (of former number) .......... | 23 |
| Do. (of the latter number) ........ | 26 |
| Total number of supposed curable cases during the year........ | 49 |

RESULTS.

| | | | | |
|---|---|---|---|---|
| Dismissed between 1st May, 1835, and 1st May, 1836, recovered, | | | | 20 |
| Do. | „ | do. | convalescent, or much improved, | 4 |
| Do. | „ | do. | somewhat improved.......... | 5 |
| Do. | „ | do. | by desire of friends, unimproved, | 2 |
| Do. | „ | do. | of incurable and inoffensive, or otherwise improper Patients | 8 |
| Dead | „ | do. | (of recent cases) ............ | 4 |
| Do. | „ | do. | (of old and confirmed cases) .. | 7 |

| | |
|---|---|
| Total number dismissed during the year ................... | 50 |
| Total number remaining in the Asylum, 1st May, 1836 ....... | 107 |

The total number of cases in which it was supposed that treatment might be serviceable, was forty-nine. Of these, it appears that twenty were dismissed quite recovered, four greatly improved, and five less improved.

" It thus appears that twenty-nine, out of the forty-nine curable Patients, have been more or less benefited by their residence in the Asylum, and already discharged; and that the number of cures is in the proportion of rather more than two-fifths of the number of those who have been placed under treatment, which is considerably above the average proportion of recoveries in most of the large public hospitals for the insane, as laid down in the statistical tables, drawn up by Esquirol, and others. And I may here also be permitted to observe, that a like proportion of recoveries has been obtained on an average of the last six years; for out of 279 Patients who have been subjected to the curative treatment of this institution, within the period just mentioned, 115 have been recovered, exclusive of thirty-three who have been dismissed, partially recovered."

It is obvious that the preceding calculation is open to a serious fallacy. More than two-fifths, says Dr. Macrobin, of those *considered* curable were cured. But what precise standard have we, to determine who are to be considered curable. At one institution, a case may be deemed quite hopeless—at another not so; and that would have the advantage in its reports, which abandoned most supinely the bad cases. The only fair mode of comparison, is that which sets the cases against the admissions. In that case, different institutions must admit all cases, curable or hopeless indiscriminately, for, if there is selection, the data for comparison are damaged or destroyed. The fact is, that in cases of insanity accurate comparisons of this sort between the results obtained at various institutions, are difficult, if not impossible.

" Of the four principal modifications or species of insanity, viz:—Mania, Monomania, Dementia or acquired fatuity, and congenital fatuity or Idiotism—the two first only are considered to be fit subjects for medical treatment; the two latter being generally believed

Dr. Macrobin dwells on these facts, in order to press on the attention of the friends of patients, and, we may add, on that of their medical attendants, the necessity of a well-timed removal from home. Dr. Macrobin also dwells on the advantages which have accrued from a well-regulated moral treatment—one calculated to engage the *attention*, and to encourage the *exercise* of the patients in the open air.

With reference to the causes which gave rise to insanity, in the different individuals who have been admitted during the year, we find that twelve of them were traced to hereditary predisposition; two to congenital imbecility; five to a highly scrofulous constitution of body; five to domestic afflictions—pecuniary embarrassments—grief, and other depressing passions; six to inebriety, and other demoralizing habits; two to an hysterical constitution, and previous attacks; two to an interruption of the uterine functions; one to injury of the head from a blow; one to child-birth; one to epilepsy; and one to a severe febrile attack; whilst two arose from occult causes, in other words from causes which could not be traced or had entirely escaped detection.

Notwithstanding, however, that such appear to have been the more prominent of the remote causes, there was unquestionably in a good many instances a combination of these in operation, all tending to the same end, some of them of a predisposing, and others of a more immediate or exciting nature. The causes of insanity are, indeed, often obscure; but in general, it will be found that several have been acting simultaneously towards its production, and also, that, in a majority of instances, there is an hereditary predisposition to it. Dr. Macrobin goes on to observe—

"That four of the female patients, who have within these last six years been discharged convalescent, have been re-admitted after the lapse of some months, or it may be one or more years, labouring under fresh attacks; but that these secondary attacks have been comparatively rare amongst the males. Females would seem, from their peculiar habits of life and situation in society, to be at all times more under the influences of the moral, than the physical causes of disease. And as it is very difficult, if not in most instances impossible, to withdraw an individual entirely from the operation of those of the former class, we are, I imagine, from this circumstance, enabled to account, in some measure at least, for the fact that they are more subject to relapses or recurrences of their complaint than males."

Eleven patients have died during the year; seven being aged and confirmed lunatics. In all the cases a post-mortem examination was instituted, and the appearances were carefully noted. Those appearances are thus generalized.

Lesions of the brain or its membranes, and sometimes of both together, were found in all of them; and although the morbid appearances in this region of the body were not in every instance precisely similar, yet were they substantially alike. Other organs of the body were also occasionally found in a state of disease. Thus five of the male patients died with symptoms of palsy and fatuity, and in all of them were discovered unequivocal traces of disease of the investing membranes of the brain, along with copious depositions of a serous fluid beneath the same, and into the ventricles. Another male patient who had long been subject to epilepsy, and who died of ileus, exhibited disease of the brain, as well as of the intestines. The last of the male patients who died, and who was in a state of fatuity when admitted, also exhibited traces of cerebral disease. The first female who died had been long subject to insanity: she exhibited marked disease of the cerebral membranes. The second was a young girl, who laboured under congenital idiotism, and whose mother has been long an inmate of the institution: she died of pulmonary consumption, and exhibited a tubercular formation in the right hemisphere of the brain; besides extensive tubercular disorganization of the lungs, peritoneum, spleen, and other abdominal viscera. The third person who died among the females had been for some time subject to violent paroxysms of mania: she also exhibited traces of cerebral disease, and two tumors were found in the cavity of the abdomen, besides intense injection of the inner coat of the stomach and bowels; the one tumor was attached to the stomach, and the other to the liver, and the irritation which they had excited in the organs with which they were in contact, appeared to have operated as the immediate cause of the maniacal paroxysms. The last was a young woman, who had been long subject to epilepsy, and was fatuous at the time of admission, and who died from exhaustion, consequent on the violent struggles which she had long sustained: the brain and its membranes were in this case much injected.

Dr. Macrobin goes on to observe:—

"In reference to this brief statement of the morbid appearances in the fatal cases, it is worthy of remark, that while five of the deaths amongst the males were preceded by a state of palsy, not one of the women exhibited this condition; and moreover, that the morbid appearances were precisely similar in all those five cases. This circumstance I have also noticed in the fatal cases of former years. I can recollect, indeed, only one instance of a female patient having exhibited symptoms of palsy during the last six years, whereas not less than a third of all the male patients who have died within the same period, had, previously to this event, fallen into the state referred to. It appears to me, that we ought to look to the differences in the nature and mode of operation of the exciting and predisposing causes, to which the two sexes respectively are most apt to be exposed, in order to obtain an explanation of this remarkable difference in the *termination* of insanity, in male and female lunatics. The males, for example, are more exposed to powerful causes of a *physical* kind, especially the abuse of spirituous liquors, tending greatly to the production of *inflammation* of the brain, and its consequences:—palsy being, together with derangement of the mental faculties, one of these consequences: while, on the other hand, the females are exposed to those *moral* causes which rather beget a state of *nervous irritation*, than of inflammation."

Those patients who are in a composed state of mind, and who are not influenced by delusions likely to be aggravated by religious exercises, are permitted to join in public worship. This moderates their passions, and is productive of very good effects.

Such is the report before us. We think it might usefully assume more of a precise and medical character. If it is not found adviseable to associate in the same report, statements adapted for the managers of the asylum and for the medical profession, Dr. Macrobin would confer a benefit upon the latter, by publishing a statistical report for it in a separate form. Some medical journal would be a good medium for it. Dr. Macrobin is evidently zealous enough to spare no pains in rendering the facts he witnesses serviceable to those who do not enjoy the same opportunities.

### Medical Statistics of the Army.*

Lieut. Tulloch, of the 45th Regiment, who is at present officially employed on the statistics of the army, has published in the United Service Journal some calculations on the mortality of officers in the army, which are interesting not only to the corps, but to all of us.

The first table which we shall present, exhibits the mortality of officers of the British army at home and abroad.

TABLE I.—P. 182.

The average annual mortality is nearly *two* per cent. throughout the whole infantry serving at home and abroad.

It may at first appear singular that the mortality should be so great among the lieutenants, who are comparatively young. The reason is a simple one—they are particularly exposed to the dangers of foreign service.

" The deaths among the lieutenants being greater than among the captains arises from the larger proportion of the former rank serving in the East Indies, where regiments have a double complement of Lieutenants. The reason of the mortality among the Majors being less than among the Captains is somewhat similar; as there is only one Major abroad with the service companies out of two, while there are six Captains out of ten. Many of the Majors are also young men, having attained that rank rapidly by purchase.

The mortality among that portion of our officers who never serve abroad, viz. the Household Troops, Heavy Cavalry, and a large proportion of the Light Dragoons, is very low. Out of an annual strength of 711, there have only died 53 in nine years, or 5·9 per annum; being in the ratio of little more than 0·8 per cent.

The mortality among the Artillery and Engineer officers who do not serve in the East Indies, and have not so large a portion of foreign service and bad stations as the Infantry, has been as follows :—

*Artillery.*—Average strength, 430—Died in nine years, 55—annually, $6\frac{1}{9}$—Ratio per cent. $1\frac{4}{10}$.

*Engineers.*—Average strength, 221—Died in nine years, 24—annually, $2\frac{6}{9}$—Ratio per cent. $1\frac{2}{10}$."

The ratio of mortality among officers on the half-pay list, according to their ranks, for the same period of nine years, is exhibited in the next table.

* British Med. Almanack, for 1837.

TABLE II.

The last table we shall admit is one which exhibits the mortality of officers in our different colonies, for the period of nine years already selected. During that period there has been comparatively little sickness in our West Indian possessions. The officers of the European corps have now considerable facilities in returning home, when in bad health, to do duty with the depôts of their corps—a circumstance which must materially tend to diminish their mortality. But the colonial corps, having no depôt companies in this country, and being generally at the worst stations, are, of course, less favourably situated, and the deaths among them are proportionally great.

TABLE III.

| STATION. | DESCRIPTION OF FORCE. | Ratio per Cent. of Deaths annually among the Officers at each Station. |
|---|---|---|
| Bengal ...... | Infantry...... | 4·7 |
| | Cavalry ...... | 2·1 |
| Madras...... | Infantry...... | 5·3 |
| | Cavalry ...... | 3·4 |
| Bombay .... | Infantry...... | 3· |
| | Cavalry ...... | 1·8 |
| Ceylon ..... | Infantry of the Line.. | 1·4 |
| | Colonial Regiments .. | 3·7 |
| | Average of all Officers .. | 2·6 |
| Mauritius .. | Infantry ...... | 1·0 |
| Windward & Leeward Islands, | Infantry of Line | 2·2 |
| | Colonial Corps | 7·1 |
| | Average of all Officers .. | 3·8 |
| Jamaica .... | Infantry...... | 3·9 |
| New S. Wales | Infantry...... | 1·2 |
| C. of Good Hope .... | Infantry...... | 1·5 |
| N. America & Bermuda | Infantry...... | ·8 |
| Gibraltar .... | Infantry...... | 1·2 |
| Malta. ...... | Infantry...... | ·9 |
| Ionian Islands | Infantry...... | 1·2 |
| Sierra Leone | Colonial Corps | 13· |
| Great Brit. & Ireland | ............. | 1·1 |

### NORTH LONDON HOSPITAL.

### RECENT OPERATIONS OF REMOVING THE SUPERIOR MAXILLARY BONE.

Our readers are aware that the superior maxillary bone has of late years been excised by many surgeons, for morbid growths originating in it. Our readers are aware also that modern experience of the effects of operations for malignant tumors and changes of structure, has tended to bring the knife into discredit, and to discountenance those frequent and bloody exhibitions which were formerly witnessed in our hospitals. It may safely be said that for ten operations for malignant growths performed twenty years ago in the hospitals, there is not more than one attempted now. A proof of two things—the ill-success of such operations in general, and the information and humanity of surgeons. The day indeed for flashy operations is gone by. The refinement of our manners is disgusted at the exhibition of what wears more the aspect of clever butchery than of science—and the amount of our experience both tells us how to prevent and when to avoid operations. Formerly the report of the performance of an operation peculiarly severe, would have excited astonishment, attracted admiration, and contributed greatly to the reputation of its performer. Now the question is instantly asked—" can that have been required—is it warrantable and judicious?"—and until that question has been satisfactorily answered, the impression is against and not for the operator. A feeling of this description has lately been excited by two reports of cases lately published in the Lancet.*

The first case was that of a young woman, 20 years of age, admitted into the North London Hospital on the 27th of September, under the care of Mr. Liston.

" Eight years since she perceived a small hard tumour projecting from the gum of the superior maxillary bone, above the second incisor tooth. It was not painful, nor did it inconvenience her. The tumour increased in size, slowly, for two months, when a medical man extracted the incisor tooth below it, which was sound. Soon after this the next tooth loosened and fell out, without being in the least decayed.—The tumour increased in size, notwithstanding the extraction of the tooth, and three years and a half since it had attained the size of a hen's egg. It was not accompanied with pain. At this time she was admitted into the *Hereford Infirmary*. In the interval, however, a surgeon had attempted to pass a lancet into the tumour, but did not succeed, on account of its hardness. While in the infirmary the tumour was partially removed, with a portion of the alveolar processes, by means of a saw and forceps. After removal it was found to present a fibrous appearance, and was very slightly ulcerated on the surface next to the lips. She continued in the infirmary for eight months, and was discharged with the wound very nearly healed, but directly afterwards the tumour began to grow again, more rapidly, and with pain."

The state of the patient on admission, was as follows:—" At present a large tumour occupies a great part of the cavity of the mouth, and projects forwards from beneath the upper lip, which is completely hidden by it. The above sketch accurately represents its external encroachments. The nose is carried upwards and to the right side by it, so that the nostrils are closed. One part of the tumour presents an ulcerated surface, covered by a thick secretion, here and there, while the other parts look red. The ulcerated surface measures, from side to side, in a line parallel to a line drawn from angle to angle of the mouth, 6½ inches, measured over the tumour. From the angle of the eye, to which the tumour extends, to a line intersecting the above, 4¼ inches. The left side of the face is much distended by the part of the tumour within the mouth, measuring, from the angle of the mouth to a line let fall perpendicularly from the ear, 5 inches. The right side of the face is less distended. Near each angle of the mouth the margin of the upper lip is stretched over the tumour, and on drawing the tumour down its continuity is rendered distinct. Within the mouth it extends on the right side, beyond the teeth, which are only seen when the tumour is drawn down by a spatula. On the left side its extent is much greater, distending considerably the integuments of the face. A groove, corresponding with the line of teeth, is observed, at the back part of which the two last molar teeth are visible. The others cannot be seen when the tumour is drawn down. In the median line it extends from the part projecting from the mouth backwards, beyond the soft palate. None of the front teeth can be seen. The colour of the tumour within the mouth is a dusky red, covered by abundant mucous secretion. The part corresponding with the palate is rather redder than the other parts. A spatula can be passed between the tumour and hard palate, on the right side. This cannot be done on the left. The integument of the face on each side is free from the tumour."

The general health of the patient was not bad. She was not aware of any cause for the complaint.

Mr. Liston decided that the disease was not malignant, because it was of long growth, of firm structure, and did not bleed except at the menstrual periods. He said "it was *certainly* not malignant," and "undertook the operation entirely on his own responsibility." That operation was thus performed.

"Mr. Liston commenced the operation by extracting the first sup. molar tooth of the right side. Then, with a stout bistoury, he made an incision from a little external to the outer angle of the left eye, in a line over the zygomatic arch, to nearly its whole extent. The second incision extended from the articulation of the frontal bone, with the ascending process of the malar bone, downwards, in a curved direction, and, guided by two fingers, passed betwixt the cheek and the tumour (the convexity of the curve being backwards) to the right commissure of the mouth. The first incision fell upon this incision, near its commencement, and at a right angle with it. The next incision extended from the left ala of the nose, in a vertical direction, through the superior lip, detaching the ala from its attachments. A similar incision was then made on the opposite side. The flaps so formed were dissected up, and fixed by an assistant, so that a large surface of the tumour was exposed. The zygomatic arch and the malar bone, near its articulation with the sphenoid and frontal, were, in succession, divided with Mr. Liston's cutting forceps. (The patient now complained of faintness, and some wine was given to her.) The nasal process of the superior maxillary bone was next divided; then the forceps were introduced so as to divide the alveolar border of the right superior maxillary bone, at the point from which the tooth had been extracted, together with the corresponding portion of the hard palate.

After the bones had been thus divided, Mr. Liston observed, that now all the difficulties were overcome, and that he had merely to turn the mass out of the cavity in which it was embedded. The patient having been refreshed by a little wine, having cleared her throat from blood, and having collected herself, the tumour was depressed gently, by the fingers resting on the lower margin of the orbit. The adhesions of the masseter, and the reflection of the membrane of the mouth on the upper lip were divided, and the diseased mass was lifted away.

The cavity of the mouth was then examined, and the soft palate found entire. Dossils of lint, moistened with water, were placed in the cavity. The flaps were retained *in situ* by a few points of twisted suture, great care being taken to adjust the margin of the lip neatly. Lint moistened with cold water was then applied over the face, and the patient conveyed to bed. During the operation, three small vessels bled smartly, but were soon checked by the finger of an assistant. No vessel required ligature."

We need not add the subsequent diurnal reports, as nothing of an untoward nature occurred. The last report is dated on the second of November, twelve days after the operation, and the patient was then walking about, had a good appetite, and her face was assuming quite a natural appearance.

On this case the Lancet offered the following observations:—

"In the report of the formidable operation which was recently executed by Mr. Liston in the *University Hospital*, for the removal of an enormous tumour of the mouth, and the bone to which it was attached, we have to record one of the most splendid triumphs which operative surgery has ever achieved. Nor was the profoundly scientific and masterly mode of accomplishing the operation the most prominent trait which the ability of the surgeon presented. The sagacity displayed by the pathologist in forming a correct diagnosis belonged, possibly, to a higher class of merit than did even the tact and dexterity which were evinced by the operator in the theatre. Eighteen months since, when the tumour was comparatively small, and the roots of its growth could be accurately traced, the sufferer was discharged, not only as incurable, but as an unrelievable object of affliction, by the surgeons of *Guy's Hospital.* The opinion of Sir Astley Cooper, the 'consulting-surgeon' of that institution, was not once solicited on the case of the unfortunate patient, so utterly destitute of hope was the miserable condition of the woman in the estimation of the surgeons of *Guy's Hospital.* Eighteen months since, she left that once-renowned institution, with a mind phrenzied by agony and despair, whereas she is now about to be restored to her friends, relieved from a mass of diseased growth, which, whilst it rendered her an object of hideous and unsightly deformity, hourly threatened her with death, in two of his most terrific forms—starvation and suffocation."

One or two observations naturally occur to the mind of the candid and well informed surgeon, on perusing this case and its attendant comments.

1. He is first tempted to inquire—are there really any means of forming such a positive opinion with respect to the malignancy or non-malignancy of a morbid growth, as Mr. Liston is said to have uttered, and seems to have acted on? If the circumstances on which he leans—slowness of growth, firmness of structure, and indisposition to fungate or to furnish serious hæmorrhage, be considered decisive evidence of non-malignancy, then there are few hospital surgeons, whose senses have not most strangely deceived them. In fact, it is well known that slow growth and a fibrous structure in a tumor are no guarantee against its cancerous nature. We may mention two cases which occur to our mind at this instant.

*a.* A boy had a fibrous tumor, which sprung from the condyloid end of the femur. It had been long in growing, and the case was thought very favourable for an operation. The thigh was amputated considerably above the tumor. That patient recovered from the operation, and died about a year afterwards with *medullary tubercles* in the lungs.

*b.* A man had a small, solid, fibrous tumor in the right side of the lower maxillary bone, near its angle. It grew from the alveolar process. The right half of the jaw was removed, much beyond the precincts of the disease. The operation did well, the wound healed, but many months afterwards, the patient returned with a large tumor of *medullary* character in the face, and symptoms of disease probably of a similar description in the lungs.

We merely mention these cases by the way. We might adduce, from published records, many like them. But it is unnecessary, because surgeons now admit that there is no precise line between malignant and non-malignant growths, which are convertible into each other, in a mode and to an extent that we are quite unable to determine. The characters alluded to by Mr. Liston are favourable it is true, but they are no more. Undoubtedly they do not carry with them the certainty that he expresses.

2. When we reflect on the capricious character of morbid growths, the readiness with which the wound made for their removal heals, and the variable period at which they re-appear, when they *do* re-appear—such excessive demonstrations of delight and confidence, as stare at us in the article in the Lancet, wear a premature and rather ridiculous aspect. The " extraordinary sagacity displayed in founding a correct diagnosis," remains yet to be proved. When two or three years have elapsed, it may be talked about. At present it is scored on trust. We may say to the reporter, as Hudibras did to Ralpho—

———— "That cuckoo's tone
Ralpho, thou always harp'st upon.
Festina lente, not too fast;
For haste (the proverb says) makes waste.
Whats'ever will not with (thy what-d'ye-call)
Thy light jump right, thou call'st synodical."

It was under these circumstances that a second case occurred, and appeared in the columns of the Lancet.

*Case.*—" W. B. aged 24, was admitted Nov. 17, under the care of Mr. Liston. Two years since he received a smart blow on the left side of the face, from a cricket-ball, which was followed by much swelling, pain and ecchymosis, all of which, however, soon disappeared. Ten months since, the last molar tooth loosened spontaneously, and was extracted, after which the gum became much swollen and tender; the swelling increased rapidly, and extended to the side of the face, and in a few weeks it attained to half of its present size. Subsequently the three teeth adjoining became loose, and were extracted. After the extraction of the second molar tooth, not many weeks ago, there was some hæmorrhage, but it was soon arrested by pressure. The growth of the tumour was rapid at first, but of late it has been less so. During its development there has not been much pain.

At present, a tumour of about the size of a very large orange, occupies the left side of the face, involving the superior maxilla. The integuments of that side are much stretched over the projecting tumour, and were of a red colour. The nose was pushed to the right side, and the lower eyelid was prevented from descending properly. Within the mouth the tumour extended backwards, in a line with the hard palate, and near to the mesial line, projecting downwards, much below the gum of the opposite side. The left nostril was completely filled, and the tumour at this part was particularly firm. The other parts of the tumour were moderately firm, and at some points elastic. All the places from which the teeth had been extracted are healed, except that from which the second molar was taken, which remains open, but is not ulcerated, nor does any fungus protrude from it. A probe can be passed a short way into the socket, but not into the tumour. The first molar tooth remains, but is loose; there is no tenderness, or pain, in the part at present.

*Operation*—Nov. 18. Previous to the patient being brought into the threatre, Mr. Liston said :—' The present operation is not one which I undertake from choice, but on account of the urgent solicitations of the patient. I do not think that the tumour is of the same benign character as the one which you lately saw removed : and although by no means so formidable-a-looking tumour, in fact, not being a sixth part of the size, yet I anticipate much greater difficulties in accomplishing its extirpation, both as regards its attachments and its vascular action; consequently, I cannot speak favourably of the ultimate result. There are, however, some points about this tumour which are not unpropitious—such as its having followed a mechanical injury—the absence of ulcerations or fungous protrusions in the situations from which the teeth have been extracted—the great firmness of the part within the nostrils, from which there has been no discharge—and our inability to pass a probe into the tumour. The removal of the growth thus early, before malignant action is developed, or the surrounding parts become contaminated, gives the patient, the only chance of its non-appearance.

The patient, on being brought into the theatre, was seated in a chair, and his head was supported on the breast of an assistant. The operation was commenced by the abstraction of the first and second incisor teeth of the left side. The first incision was commenced over the malar prominence, the operator cutting down to the bone, and through the integuments into the mouth, continuing the incision, in a curved line, to the left commissure of the mouth, the convexity of the curve being backwards. The second incision extended along the side and alæ of the nose, to the median line of the lip. The flaps so formed were dissected up from the tumour, which was exposed, together with part of the malar bone. The malar bone was now cut through, near to the superior maxilla, with Hey's saw, and the ascending nasal process of the superior maxillary bone was divided with the cutting forceps. With the same instrument, a notch was cut out from the border of the superior maxillary bone, at the point from which the teeth were extracted; and the hard palate was divided along, near the mesial line, with a strong pair of scissars, to the palate bone; with the left hand, the tumour was depressed, and the remaining adhesions in the orbit were divided with the knife. The mass was then removed with great facility. The cavity having been sponged out, some of the morbid growth was found adhering to the ascending plate and cell of the palate bone, and slightly to the pterygoid process of the sphenoid bone. This was removed by cross-cutting forceps. The cavity was again sponged out, and, upon a careful examination, no more of the morbid growth was found remaining, or any vessel discovered bleeding. It was then filled with dry lint, and the flap brought down, and fixed by means of two twisted and interrupted sutures."

The patient was conveyed to bed after the operation, much exhausted, with rapid pulse, cold extremities, and occasional convulsions. He never rallied afterwards, and, at 11, p.m. of the day of the operation he expired, evidently from the effects of the operation.

"*The tumour* was an albuminous sarcoma, consisting of cartilage, with spiculæ of bone, and a glairy matter coagulable by heat. On examining the cavity, a clot of blood was found in it, upon the lint. The whole of the palate bone, and the root of the pterygoid process, were removed, leaving the internal and external pterygoid process with the soft palate. The sphenoidal cells were entire, the opening into which was easily seen, with a well-defined margin, covered with its proper lining membrane. A small portion of the ethmoid was removed. The cells of this bone, together with those of the sphenoid cells, were quite free from disease. No further *post-mortem* was allowed.

Since the death of the patient, it has been ascertained that he had led a most dissolute life, which, before the operation, he most strongly and repeatedly denied."

To this case, itself of no ordinary complexion, the most extraordinary comments are appended. Before we give them we will make a remark or two.

The reporter took occasion to laud Mr. Liston for his sagacity in the diagnosis of the preceding case. It was not simply that he operated wonderfully well, his sagacity in determining that the disease was not malignant, was more wonderful still. But, if the diagnosis in that instance was worth any thing, what shall we say to it in this? In that case, the surgeon operated because the disease was of slow growth, of firm texture, and did not bleed. In this case, he operates when the disease is of rapid growth, of unequal texture, and *does* bleed! If the decision was right in one instance, it is clearly wrong in the other, and the "sagacity" displayed is of a very contradictory character.

Two circumstances are urged in extenuation, for that appears to be thought necessary, of the operation. First, we are told that it was done at the solicitation of the patient. Supposing the operation to be improper, that is no excuse at all, but the most damnatory circumstance that could be adduced against the surgeon. *He* is the arbiter of the propriety of the operation—to *his* judgment the patient confides his life in trust—on *him* it rests to say what should or should not be done. If this is true of operations in general, how true must it be of such an operation as this—an operation so disgusting and revolting in appearance, that an eye-witness declares that, of "upwards of two hundred spectators present, many became faint, and some were carried out of the theatre—such was the scene"* of an operation founded on the diagnosis

* Lond. Med. and Surg. Jour. No. 254.

of a morbid growth, of which a patient must of course be ignorant—an operation attended with urgent risk, and liable to be followed, as the event proved, with speedy death.

The intelligent reader need not be told, that there are not merely two parties in a question of this nature—but that, besides the interest of the patient and the reputation of the surgeon, the wider interests of humanity and the character of science are involved. An unsuccessful operation, particularly if its details are bloody and disgusting, an unsuccessful operation, even if warranted by the soundest judgment, exerts a prejudicial effect on the community. The timid are alarmed, the cautious are shaken, and that one operation which has failed, will probably prevent many from submitting to operations which would prove successful. A doubt is thrown, both on the discrimination and the humanity of surgeons, and the vulgar may be excused for harbouring the prejudice, that opportunities for displaying brute force and sleight of hand are not always repudiated, when a life or a limb is on the issue.

The second reason for operating to which we shall allude, is founded on the tumor having followed a blow. A singular reason, because every body knows that malignant growths very frequently originate in slight local injuries. In fact when a local injury is followed by a morbid growth quite disproportioned to the amount of injury, that is presumptive evidence for, rather than against, constitutional vitiation.

The history of the case, the characters of the tumor, its examination when removed, all concur to prove that the disease *was* malignant, and the patient's rapid death, shocking as it may appear, was really a boon to him. Had he recovered from the immediate effects of the knife, he would in all probability have been reserved for a fate more lingering and more miserable—a return of the disease, with all *its* horrors.

We shall now extract the comments of the reporter, to which we have referred.

"Although the result of the operation performed in the above case, was unfortunate with regard to the suffering patient, it furnishes a useful lesson of admonition to students and young practitioners. Often does it happen that a surgeon who has just emerged from the portals of the college, seeks for opportunities to display his skill in operating, that he may thus establish his reputation as an operating surgeon. Is it not evident from the report of the case before us, that the first attempt of the juvenile aspirant might be made on a patient whose constitution, weakened and broken down by dissipation, could not recover from the shock thus inflicted upon his feeble powers? Ever should it be recollected, then, by young practitioners, that success after operations cannot be *ensured*. Advantageous as must be the result of this case to the student, and to the youthful members of the profession generally, it must prove of still greater value to Mr. Liston himself. The reputation of that gentleman as an operator stands unrivalled, and the dexterity which he possesses is a subject of astonishment with surgeons who have visited the continental hospitals. The public, therefore, on discovering that an operation may occasionally be followed by fatal consequences, even when it is performed by the most distinguished of our surgeons, will shrink in dismay from the thought of entrusting their lives, in diseases requiring operations, to half-instructed bunglers, who, under the pernicious influence of the system of nepotism, obtain the office of surgeon in our old endowed hospitals. The issue of the case of the patient, E. B., proves beyond question or dispute, that capital operations in surgery cannot be undertaken, with safety to the reputation of the practitioner, unless by such a man as Mr. Liston,—a surgeon of undoubted skill and established fame."

A more bungling attempt to protect a friend could scarce be made. From such friends Mr. Liston may pray Heaven to defend him. It would be difficult to string together a more gross collection of truisms and non-sequiturs, or having strung them, to lay on a thicker coat of the most dingy varnish.

Yet there is some ingenuity too. By a hocus-pocus process, the failure of Mr. Liston is made a proof of his consummate ability and a proof too of the ignorance of other people. He performs an operation, which probably, under the circumstances, no other London hospital surgeon would attempt. The patient dies from its effects. Ergo, those surgeons are "half-instructed bunglers," and he is a man of "undoubted skill." To be sure, we should be grateful for the novel information, that "success after operations cannot be ensured." That is a discovery which was certainly reserved for the reporter; yet withal, it may be doubted whether the "students and young practitioners" are quite such simpletons as to be led by the nose, although he so obligingly soaps it for them.

The climax of his reasoning, is reserved by the reporter for his concluding paragraph. E. B. being operated on by Mr. Liston, dies in consequence of the operation—*therefore*, the issue of E. B's case proves that capital operations cannot be undertaken with safety to the reputation of the operator, unless by such a man as, Mr. Liston!

In one sentiment we quite coincide—that this case will be of value to Mr. Liston himself. No doubt it will. 'Once singed, twice shy,' is a jockey's proverb.

We have noticed these cases at some little length, because they have been much talked of, and because they bear on a question of much interest and of vital consequence—the propriety of performing serious and dangerous operations for morbid growths. We have seen in our time a growing disinclination on the part of surgeons to perform, and on that even of students to witness, operations of doubtful utility. It is an honourable and a scientific trait in both, and is a part of that spirit, which happily is prevailing more and more, of avoiding the infliction of suffering both on man and brute, and respecting human life. We see its operation in the mitigation of penal codes—in the diminished frequency and milder laws of war—we see it even in popular revolutions—and, within the pale of our own body, we see it in the comparative disuse of the knife;—may that be less and less resorted to.

